# Sanren decoction ameliorates ulcerative colitis by modulating gut microbiota and macrophage polarization to enhance intestinal barrier function

**DOI:** 10.1186/s13020-025-01183-1

**Published:** 2025-08-20

**Authors:** Shujuan Zhang, BangHua Li, Xiaoqun Han, LinLin Ruan, Xingxing Fu, Yanglin Chen, Hongtao Wan, Xiaojian Zhu, Dan Liu, Bo Yi

**Affiliations:** 1 2Nd Abdominal Surgery Department, Jiangxi Cancer Hospital, The Second Affiliated Hospital of Nanchang Medical College, Nanchang, 330029 Jiangxi People’s Republic of China; 2 Department of Integrated Traditional Chinese and Western Medicine Oncology, Third Ward of Gastrointestinal Oncology. School of Pharmacy, Jiangxi Medical College, Nanchang University, Nanchang, 330006 People’s Republic of China; 3 School of Chemistry and Bioengineering, Yichun University, Yichun, 336000 People’s Republic of China; 4 School of Pharmacy, Jiangxi Medical College, Nanchang University, Nanchang, 330006 People’s Republic of China; 5 Department of Pharmacy, Jiangxi Cancer Hospital, The Second Affiliated Hospital of Nanchang Medical College, Nanchang, 330029 Jiangxi People’s Republic of China; 6 Jiangxi Key Laboratory of Oncology, Jiangxi Cancer Hospital, Nanchang, 330006 People’s Republic of China

**Keywords:** Sanren decoction, Ulcerative colitis, Gut microbiota, PTGS2, Macrophage polarization, Microbial dysbiosis

## Abstract

Sanren Decoction (SRD), a traditional Chinese medicine formula historically used to relieve damp-heat and gastrointestinal disorders, demonstrates targeted efficacy in ulcerative colitis (UC)—a disease marked by chronic inflammation and mucosal damage—by acting on multiple pathological pathways. This study employed an integrative methodology comprising network pharmacology, molecular docking, and in vivo experimentation to elucidate the underlying pharmacological mechanisms of SRD in the management of UC. The findings identified 87 genes targeted by SRD that are associated with UC, highlighting PTGS2 (Prostaglandin-Endoperoxide Synthase 2) as a crucial target involved in inflammatory processes. Molecular docking analysis confirmed significant interactions between the active compounds of SRD and PTGS2, suggesting a potential anti-inflammatory pathway. In vivo experiments utilizing a DSS-induced colitis mouse model demonstrated that SRD effectively ameliorates clinical symptoms and histopathological damage, enhances intestinal barrier integrity, and modulates macrophage polarization from a pro-inflammatory M1 state to an anti-inflammatory M2 phenotype. Furthermore, SRD was found to alter gut microbiota composition by increasing the abundance of beneficial bacteria and influencing metabolic pathways. These findings establish a strong scientific foundation for the potential of SRD as a comprehensive therapeutic approach for UC, offering promising prospects for its integration into clinical practice.

**Supplementary Information:**

The online version contains supplementary material available at 10.1186/s13020-025-01183-1.

## Introduction

Ulcerative colitis (UC) is a chronic inflammatory disease of the colon and rectum, characterized by symptoms such as abdominal pain and diarrhea [1, 2]. Its pathogenesis is multifactorial, involving genetic [3], environmental, immune, and gut microbiota factors [4–6]. While current treatments can alleviate inflammation and induce remission, they are often associated with significant side effects in many patients [7, 8]. Additionally, some patients develop resistance, highlighting the need for alternative therapies [9, 10]. Traditional Chinese Medicine (TCM), specifically Sanren Decoction (SRD), has shown promise in managing UC due to its anti-inflammatory, immunomodulatory, and gut microbiota-regulating properties. Several recent clinical studies have reported that SRD or its modified forms significantly improve symptoms and reduce inflammation in UC patients [11–13]. However, its specific molecular mechanisms and therapeutic targets remain unclear.

A crucial aspect of UC progression is the polarization of macrophages in the intestinal immune response [14]. Macrophages can differentiate into either pro-inflammatory M1 or anti-inflammatory M2 phenotypes [15]. M1 macrophages contribute to the amplification of inflammation and tissue damage, while M2 macrophages play a role in promoting tissue repair and regeneration [16, 17]. Modulating macrophage polarization towards the M2 phenotype could offer a therapeutic approach for UC. Recent findings suggest that SRD may influence this polarization by reducing M1 activity and enhancing M2 function, potentially contributing to its therapeutic effects.

To explore the multi-target mechanisms of herbal medicines like SRD, network pharmacology and molecular docking are invaluable. Network pharmacology can predict interactions between active compounds and biological targets, mapping out the complex pathways involved in disease modulation [18]. Molecular docking further validates these interactions at the molecular level by demonstrating binding affinities between compounds and target proteins [19]. Additionally, animal models, such as dextran sulfate sodium (DSS)-induced colitis in mice [20], enable the in vivo assessment of the effects of SRD on inflammation, intestinal barrier function, and gut microbiota composition.

The gut microbiota plays a crucial role in the development and progression of UC, a chronic inflammatory condition of the colon [21]. Dysbiosis, characterized by an imbalance in the composition of the gut microbiota, has been closely linked to promoting intestinal inflammation, impairing mucosal barrier integrity, and disrupting overall mucosal homeostasis [22, 23]. This imbalance contributes to the pathogenesis of UC by enhancing immune dysregulation and increasing susceptibility to inflammation. Given the pivotal role of gut microbiota in UC, strategies aimed at modulating its composition and function have emerged as promising therapeutic approaches. Investigating how SRD influences gut microbiota and its associated metabolites is crucial for elucidating the mechanisms underlying its potential therapeutic efficacy in managing UC.

Although the potential of SRD is recognized, research integrating network pharmacology, molecular docking, macrophage polarization, and animal studies to elucidate the mechanisms underlying UC comprehensively has been limited. Most research has concentrated on the general anti-inflammatory properties, with limited exploration of its multi-target effects or its influence on macrophage polarization and gut microbiota dynamics. This gap limits the optimization of SRD for clinical use in UC treatment. Therefore, this study aims to systematically investigate the pharmacological mechanisms of SRD in UC through an integrative approach. Network pharmacology will be employed to identify potential therapeutic targets and pathways, with molecular docking used to validate these interactions. The DSS-induced colitis model in mice will be utilized to assess the in vivo efficacy of SRD, focusing on its impact on clinical symptoms, histopathology, inflammatory cytokines, macrophage polarization, intestinal barrier integrity, and gut microbiota composition. This comprehensive analysis aims to provide a deeper understanding of the therapeutic effects of SRD in UC, thereby establishing a scientific foundation for its clinical application and contributing to the development of more effective treatment strategies for this chronic disease.

## Materials and methods

### Reagents

IL-6 Detection Kit (Nanjing Bioyntech, BY-EM220188); TNF-α Detection Kit (Nanjing Bioyntech, BY-EM220852); IL-1β Detection Kit (Nanjing Bioyntech, BY-EM2201740); β-Actin Monoclonal Antibody (Proteintech, 66009-1-Ig, 1:2000); Occludin Polyclonal Antibody (Proteintech, 27260-1-AP, 1:2000); ZO-1 Polyclonal Antibody (Proteintech, 21773-1-AP, 1:2000); Claudin-5 Polyclonal Antibody (Proteintech, 29767-1-AP, 1:2000); Anti-COX2/Cyclooxygenase 2 Rabbit mAb (PTMBIO, PTM-7147, 1:1000); Anti-iNOS antibody[RM1017] (abcam, ab283655, 1:2000); Arginase-1 Polyclonal antibody(Proteintech,1600–1-AP, 1:5000); GAPDH Rabbit mAb (zenbio, R380626, 1:5000); Anti-beta Tubulin Rabbit pAb (Servicebio,GB11017,1:2000); CD86 (SAB, 48763-1, 1:400); CD163 (Proteintech, 16646-1-AP, 1:400); F4/80 (Zenbio, 263101, 1:400); MUC2 Ready-to-use Reagent Kit for IHC (Proteintech, KHC1327, 1:1200); Goat Anti-Mouse IgG H&L (UE, H6161, 1:8000); Goat Anti-Rabbit IgG H&L (UE, H6161, 1:8000); Two-Step Immunohistochemistry Detection Kit (BOSTER, SV0002); DyLight 594 Conjugated AffiniPure Goat Anti-Mouse IgG H&L (BOSTER, BA1141, 1:400); iFluor™ 488 Conjugated Goat Anti-Rabbit IgG Polyclonal Antibody (HUABIO, HA1121, 1:400); Ampicillin (TargetMol, T0814L); Vancomycin (TargetMol, T8641); Neomycin Sulfate (TargetMol, T0950); Metronidazole (TargetMol, T1079);DSS for dextran sulfate sodium salt colitis modeling (meilunbio, MB5535-2);16S rRNA and untargeted metabolomics sequencing was provided by Metware Biotechnology Co., Ltd. (Wuhan, China); Mice were purchased from Jiangsu Jicui Yaokang Biotechnology Co., Ltd. (Nanjing, China); The mice were housed at the Laboratory Animal Science Center of Nanchang University (Nanchang, China).

### Network pharmacology

This study utilized a network pharmacology approach to analyze the active components of SRD. Constituents from Coix seed, Arum Ternatum Thunb, Amygdalus Communis Vas, Tetrapanacis Medulla, Alpinia katsumadai Hayata, and Magnolia Officinalis Rehd Et Wils were retrieved from the TCMSP database, with criteria set at oral bioavailability (OB) ≥ 30% and drug-likeness (DL) ≥ 0.18. Targets for Lophatherum gracile and Pulvis Talci were identified using the BATMAN-TCM database (score ≥ 2.0, *P* ≤ 0.05) and validated against Uniprot to eliminate duplicates. UC-related genes were obtained from GeneCards. Venn diagrams comparing UC-related genes and SRD targets were created on the Xiantao Academic platform. Drug-active component and component-disease target networks were constructed in Cytoscape (v3.8.0), and a protein–protein interaction (PPI) network was developed using STRING to identify the top 10 core proteins. KEGG pathway enrichment analysis was performed via Metascape.

### Preparation of SRD and UPLC-MS/MS analysis

The SRD was prepared from a specific formulation comprising 18 g of raw Coicis Semen, 15 g of Bitter Apricot Kernel, 6 g of Amomum Cardamomum Fruit, 6 g of Magnoliae Officinalis Cortex, 15 g of Pinelliae Rhizoma Praeparatum, 6 g of Lophatherum Herba, 18 g of Talcum, and 6 g of Tetrapanacis Medulla, more information on herbs is provided in Table 1, all obtained from Jiangxi Provincial Cancer Hospital. The herbs underwent a thorough process of pulverization, decoction, and filtration, yielding an extract that was concentrated to a final density of 2.0 g/mL using a rotary evaporator.

**Table 1:** Composition of Sanren decoction(SRD)

NO	Chinese name	Latin name	Part used	Dose(g)
1	Yiyiren	Coicis Semen	Seed	18 g
2	Banxia	Arum Ternatum Thunb	Tuber	15 g
3	Kuxingren	Amygdalus Communis Vas	Seed	15 g
4	Tongcao	Tetrapanacis Medulla	Stem	6 g
5	Kouren	Alpinia katsumadai Hayata	Seed	6 g
6	Huashifen	Pulvis Talci		18 g
7	Houpu	Magnolia Officinalis Rehd Et Wils	Bark	6 g
8	Danzhuye	Lophatherum gracile Brongn	Leave	6 g

### UPLC-MS/MS analysis

UPLC-MS/MS data for SRD were collected using a Vanquish UPLC system and Q Exactive HFX mass spectrometer (Thermo, USA), employing a Waters HSS T3 column (100 × 2.1 mm, 1.8 μm) with a mobile phase of 0.1% formic acid in water (solvent A) and acetonitrile (solvent B) at a flow rate of 0.3 mL/min, column temperature of 40 °C, and 2 μl injection volume. Spectra were collected in positive and negative ion modes, with a scan range of 70–1050 Da and resolutions of 70,000 (first level) and 17,500 (second level). Raw data were processed using Progenesis QI for baseline filtering, peak recognition, and alignment, resulting in a matrix of retention time, mass-to-charge ratio, and peak intensity. Component identification was supported by databases such as HMDB and METLIN.

### Molecular docking

A total of 35 active components were identified through UPLC-MS/MS and docked with the key factor PTGS2. The two-dimensional structures of these components were retrieved from the PubChem database (https://pubchem.ncbi.nlm.nih.gov/), and the protein structure of the target gene was obtained from RCSB (https://www.rcsb.org/). The protein was preprocessed using MOE software (2022.02), followed by docking of the protein with the small molecules, resulting in interaction diagrams of the active molecules and the protein.

### Animal experiment design

#### Preliminary experiment

Six-week-old male C57BL/6 J mice, sourced from Jiangsu Jicuiyaokang, were housed under specific pathogen-free conditions at the Experimental Animal Science Center of Nanchang University. The study was approved by the Nanchang University Ethics Committee (NCULAE-20240520002). A preliminary experiment was conducted to determine the optimal dose of SRD for the treatment of UC (Fig. 4A). Colitis was induced in the mice using a 2.5% DSS solution, after which SRD was administered orally at low (0.092 mL/animal/day), medium (0.184 mL/animal/day), and high (0.368 mL/animal/day) doses over 14 days. Four experimental groups were established: DSS only, low-dose SRD, medium-dose SRD, and high-dose SRD. Body weight, diarrhea, and rectal bleeding were monitored daily. Upon the conclusion of the experiment, the mice were anesthetized with 1.5–2% isoflurane by inhalation and maintained at a stable body temperature of 37 °C ± 0.5 °C. Serum samples were subsequently collected for the quantification of IL-1β via ELISA, and the colon tissues were subjected to histopathological analysis. The medium dose exhibited optimal therapeutic efficacy and was selected for further investigation.

#### Establishment of the mouse UC model

Twenty-eight male C57BL/6 J mice, aged 6–8 weeks, were acclimated for one week and divided into four groups: control (n = 6), DSS (n = 7), positive control treated with sulfasalazine (200 mg/kg/day) (n = 7), and SRD treatment group (n = 8). For the treatment duration from day 8 to day 14, all groups except the control received a 2.5% DSS solution daily to induce colitis(Fig. 4E). Mice in the SRD group were treated orally with the selected medium dose of SRD.

#### Fecal microbiota transplantation (FMT)

FMT was performed to investigate the effects of SRD on gut microbiota, following the methods established in a previous study [24]. Thirty male C57BL/6 J mice were divided into Control and SRD donor groups, receiving 0.184 mL/animal/day SRD or saline via oral gavage for 28 days. Fecal samples were collected from days 15 to 28, homogenized with sterile saline, and centrifuged. The supernatant was administered to recipient mice via oral gavage. Twenty-four recipient mice were pre-treated with an antibiotic cocktail for 7 days to deplete their gut microbiota, followed by a 48 h washout period. From days 15 to 28, they received daily gavage of fecal microbiota from Control or SRD donors. Each mouse was administered 200 μL of supernatant, which was prepared within 10 min prior to administration. Colitis was induced from days 21 to 28 using a 2.5% DSS solution (Fig. 9A). After treatment, the mice were euthanized, and their colon tissues and contents were collected for analysis.

### Disease activity index (DAI) evaluation

The severity of colitis was assessed daily by monitoring body weight, fecal bleeding, and stool consistency. The Disease Activity Index (DAI) was calculated using the formula: DAI = (Weight Loss Score + Fecal Occult Blood Score + Diarrhea Score)/3, providing a quantitative measure of disease progression and treatment response [25]. Weight change: 0, no weight loss; 1, 1–5% weight loss; 2, 5–10% weight loss; 3, 10–15% weight loss; 4, > 15% weight loss. Stool consistency: 0, normal; 2, dilute stool; 4, diarrhea. Blood in stool: 0, no blood; 2, pellet bleeding; 4, severe bleeding and blood around the anus.

### Enzyme-linked immunosorbent assay (ELISA)

Inflammatory cytokine levels were measured by first rinsing the colonic tissues with pre-cooled phosphate-buffered saline (PBS) to remove fecal matter. The tissues were then weighed, minced, and mixed with PBS in a 1:9 ratio. The tissue-PBS mixture was subjected to ultrasonic disruption at 60 Hz for 1 min, repeated 2–3 times. After homogenization, the mixture was centrifuged at 5000 r/min for 20 min, and the supernatant was collected for ELISA analysis. Cytokines TNF-α, IL-1β, and IL-6 were quantified following the manufacturer’s instructions for the reagents.

### Western blotting (WB)

Frozen colon tissue (20 mg) was lysed in a 1:9 ratio of lysis buffer, homogenized, and centrifuged at 12,000 rpm for 10 min at 4 °C to collect the protein supernatant. After adding the loading buffer, the sample was heated. The proteins were then transferred to PVDF membranes and incubated with primary antibodies: ZO-1 (1:1000), Occludin (1:1000), Claudin-5 (1:1000), PTGS2 (1:1000), iNOS (1:2000), ARG1 (1:5000), β-actin (1:4000), GAPDH (1:5000) and Tubulin (1:2000) followed by incubation with secondary antibodies. The results were analyzed using ImageJ software.

### Histological analysis

Colon tissues fixed in paraformaldehyde were embedded in paraffin, sectioned at 4 μm, and stained with hematoxylin and eosin (H&E) and alginate blue-periodic acid-Schiff (AB-PAS), following the protocols provided by the reagent kits (Solarbio, Beijing, China).

### immunohistochemistry (IHC)

The immunohistochemical method was used to detect the expression changes of inflammatory mediator proteins in the colon tissues of mice in each group. Paraffin blocks embedded in colon tissues were sliced, deparaffinized, antigen repaired, blocked, and incubated with primary antibody (MUC2 Ready to use Reagent Kit for IHC), secondary antibody (Two Step Immunohistochemistry Detection Kit), DAB staining, counterstaining of cell nuclei, neutral gum sealing, microscopic examination, and observation under an optical microscope. The cell nucleus in the image appears blue, while the positive expression appears brownish-yellow.

### Immunofluorescence

Tissue samples were fixed in 4% paraformaldehyde, embedded in paraffin, sectioned, and then deparaffinized. Endogenous peroxidase activity was blocked with 3% H₂O₂ for 10 min. Following PBS washes and antigen retrieval, sections were blocked with 5% BSA at 37 °C for 30 min and incubated overnight at 4 °C with the primary antibody. The next day, sections were incubated with a secondary antibody for 2 h in the dark. DAPI staining was conducted for 5 min, and samples were mounted with an anti-fade medium for visualization under an inverted fluorescence microscope(Leica GmbH, Germany).

### 16S rRNA sequencing

Fecal samples from mice, stored at − 80 °C, were processed for 16S rRNA sequencing according to established protocols [26]. DNA was extracted using commercial kits, following the manufacturer's instructions, and the V3-V4 region of the bacterial 16S rRNA gene was amplified. The resulting amplicons were purified and sequenced on the NovaSeq 6000 platform. Operational taxonomic units (OTUs) were identified using the Uparse algorithm (USEARCH v7) with a sequence similarity threshold of 97% or higher. Alpha diversity, including the Shannon, Simpson, and Chao1 indices, was calculated using R (v4.1.2) and QIIME (v1.9.1). Beta diversity was assessed through Non-Metric Multidimensional Scaling (NMDS), Principal Component Analysis (PCA), and Principal Coordinates Analysis (PCoA). The gut microbiota composition was further analyzed using Linear Discriminant Analysis Effect Size (LEfSe), with significantly enriched taxa identified based on an LDA score threshold of greater than 4. The 16S rRNA sequencing data has been uploaded to https://submit.ncbi.nlm.nih.gov/subs/sra/ with the serial number PRJNA1173098

### Untargeted metabolomics

The samples, stored at −80 °C, were thawed on ice. To each sample (20 mg), 400 μL of internal standard solution (methanol: water = 7:3, v/v) was added, and the mixture was vortexed for 3 min. The samples were sonicated in an ice bath for 10 min, vortexed for 1 min, and sonicated again at − 20 °C for 30 min. After centrifugation at 12,000 rpm for 10 min at 4 °C, the supernatant was collected. The pellet was centrifuged again at 12,000 rpm for 3 min at 4 °C, and 200 μL of the supernatant was transferred for LC–MS analysis by Metware Biotechnology Co. (Wuhan, China). Metabolites were identified using the KEGG compound database (http://www.example.com/compound/) and mapped to the KEGG Pathway database (http://www.kegg.jp/kegg/pathway.html) for pathway enrichment analysis. Correlation heatmaps were generated using hierarchical clustering in the ComplexHeatmap package in R. Spearman’s correlation analysis was conducted to examine the relationship between differential microbiota and metabolites.

### Statistical analysis

All data were statistically analyzed using GraphPad Prism 8.0. The differences between the two groups are tested using an unpaired *t*-test. The differences between multiple groups were identified by one-way analysis of variance (ANOVA). *P* values < 0.05 were considered to be statistically significant.

## Results

### Identification of potential SRD targets for UC inhibition through network pharmacology and molecular docking analysis

**Fig. 1:**
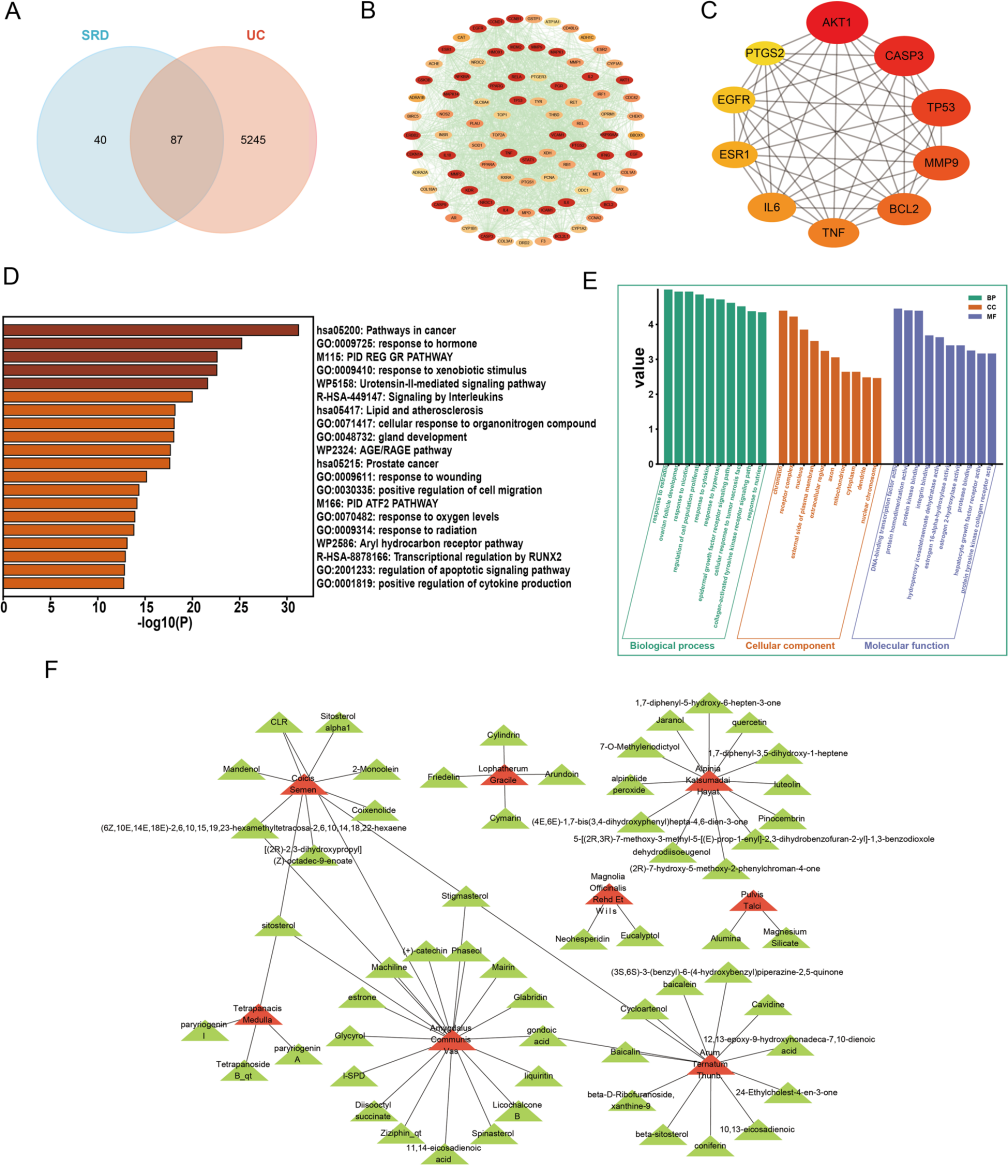
Network pharmacology analysis of Sanren decoction (SRD). **A** Venn diagram illustrating the shared targets between SRD and UC. **B** Protein–protein interaction (PPI) network of shared targets between SRD and UC. **C** The top 10 core targets in the PPI network. **D** Kyoto Encyclopedia of Genes and Genomes (KEGG) enrichment analysis. **E** GO enrichment analysis of SRD-related targets. **F** Network diagram of the drug and active components

**Fig. 2:**
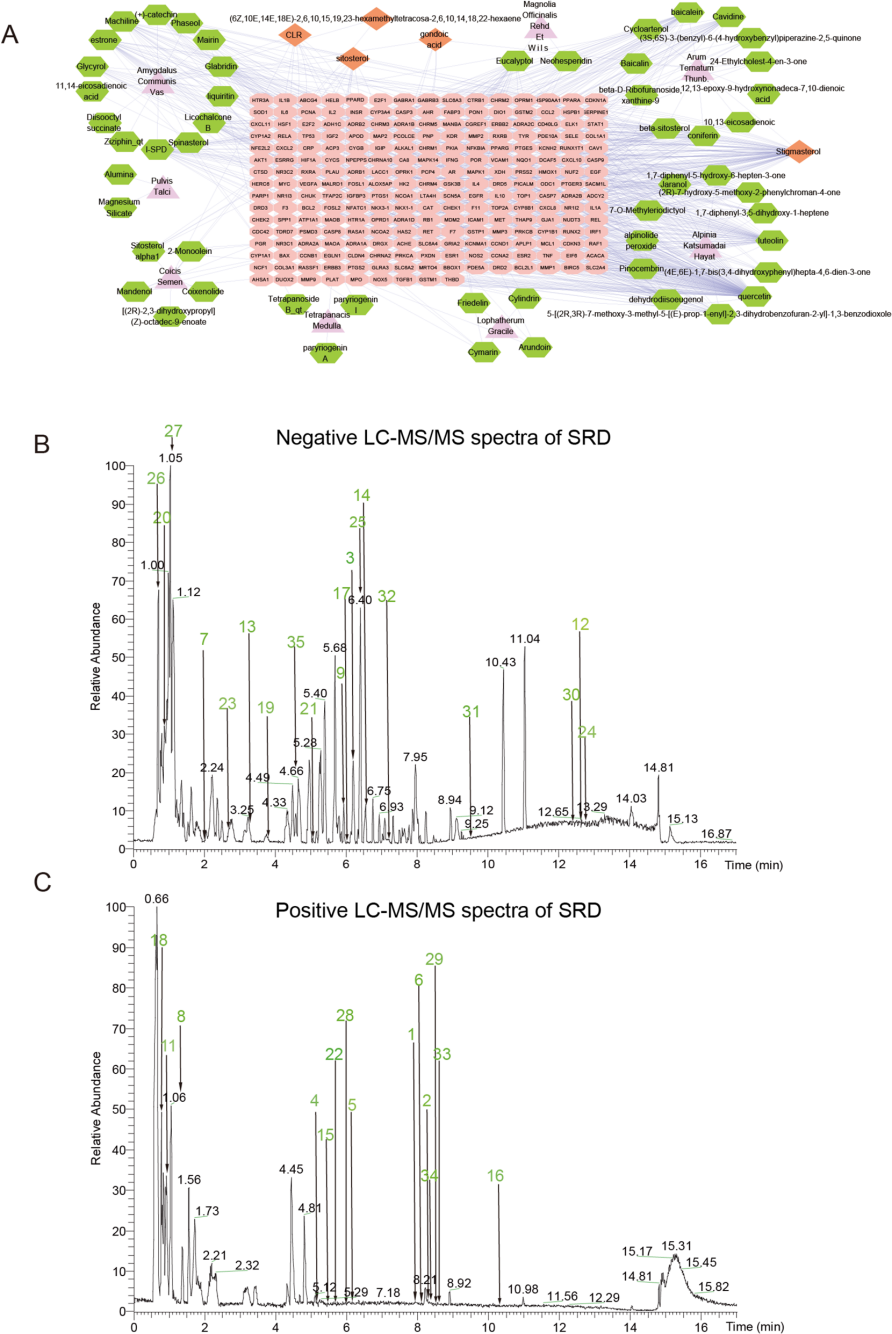
Network pharmacology and LC–MS/MS spectral analysis of SRD. **A** Network illustrating the active components and their associated disease targets. **B** Negative ion mode spectra of SRD. **C** Positive ion mode spectra of SRD

To elucidate the pharmacological mechanisms underlying Sanren Decoction (SRD) in the treatment of ulcerative colitis (UC), we employed a network pharmacology approach. By integrating the predicted targets of SRD's active compounds with genes associated with UC pathology, 87 common target genes were identified (Fig. 1A). A protein–protein interaction (PPI) network was constructed to map the interactions among these targets, highlighting highly connected hub genes in red, which indicates their central role in both UC pathogenesis and SRD's therapeutic mechanism (Fig. 1B). Among the top 10 core targets, PTGS2 emerged as a pivotal target due to its well-documented role in inflammatory processes (Fig. 1C). Pathway enrichment analysis revealed that 66 common targets were significantly involved in the top 20 Kyoto Encyclopedia of Genes and Genomes (KEGG) pathways (Fig. 1D). These pathways are closely related to inflammation and disease processes, including cancer-related pathways and interleukin signaling. To complement KEGG analysis, Gene Ontology (GO) enrichment was performed. The results revealed enrichment in biological processes such as cytokine response and epithelial development, as well as molecular functions like cytokine receptor binding and kinase activity (Fig. 1E), highlighting SRD’s multi-pathway immunoregulatory potential. Additionally, drug-active component and active component-disease target networks were constructed using Cytoscape (Figs. 1F, 2A). LC–MS/MS analysis identified 35 compounds in SRD, with comprehensive scores above 90, including flavonoids, terpenoids, and organic compounds (Fig. 2B, C; Table 2).

**Table 2:** Total active ingredients of SRD

Serial number	Mode	Metabolite	Fragmentation Score	Formula	m/z
1	pos	Diosmetin	98.6	C16H12O6	301.0709316
2	pos	Glycyrrhizic acid	98.5	C42H62O16	823.4123162
3	neg	Salicylic acid	97.9	C7H6O3	137.0247626
4	pos	Isoorientin	97.2	C21H20O11	449.1086015
5	pos	Diosmetin-7-O-Beta-D-glucopyranoside	96.2	C22H22O11	463.1239408
6	pos	Tectorigenin	95.3	C16H12O6	301.0710482
7	neg	Calcium pantothenate	95		218.1041225
8	pos	ADENOSINE	94.9	C10H13N5O4	268.1044195
9	neg	Vanillin	94.5	C8H8O3	151.0406106
10	pos	Orientin	94.5	C21H20O11	449.1085545
11	pos	Guanine	94.5	C5H5N5O	152.0570099
12	neg	Usniacin	93.8	C18H16O7	343.0835727
13	neg	Protocatechuic acid	92.9	C7H6O4	153.0199174
14	neg	Coniferaldehyde	92.9	C10H10O3	177.05638
15	pos	Liquiritigenin	92.8	C15H12O4	257.0811132
16	pos	Sericic acid	92.7	C30H48O6	469.332524
17	neg	scopoletin	92.5	C10H8O4	191.0357622
18	pos	D-proline	92.5	C5H9NO2	116.0710147
19	neg	Forsythoside E	92.3	C20H30O12	461.1676195
20	neg	Citrate	92.1	C6H8O7	191.0203369
21	neg	Vicenin III	91.8	C26H28O14	563.1443849
22	pos	Andrographiside	91.8	C26H40O10	495.2593971
23	neg	Benzyl gentiobioside	91.4	C19H28O11	431.1576091
24	neg	Scillarenin	91.4	C24H30O4	381.2060002
25	neg	Coixol	91.4	C8H7NO3	164.0359302
26	neg	Raffinose	91.4	C18H32O16	503.1622072
27	neg	L-Tyrosine	91.4	C9H11NO3	180.0671842
28	pos	Suavioside A	91.4	C26H44O8	526.3381157
29	pos	formononetin	91.4	C16H12O4	269.0811691
30	neg	Glycyrrhetinate	90.8	C30H46O4	469.3336975
31	neg	6-Gingerol	90.8	C17H26O4	293.1762804
32	neg	rhapontin	90.2	C21H24O9	419.1359806
33	pos	Juncuenin D	90.2	C18H18O3	265.1227326
34	pos	Torilolone	90.1	C15H24O3	235.1696075
35	neg	amygdalin	90	C20H27NO11	456.1524036

**Fig. 3:**
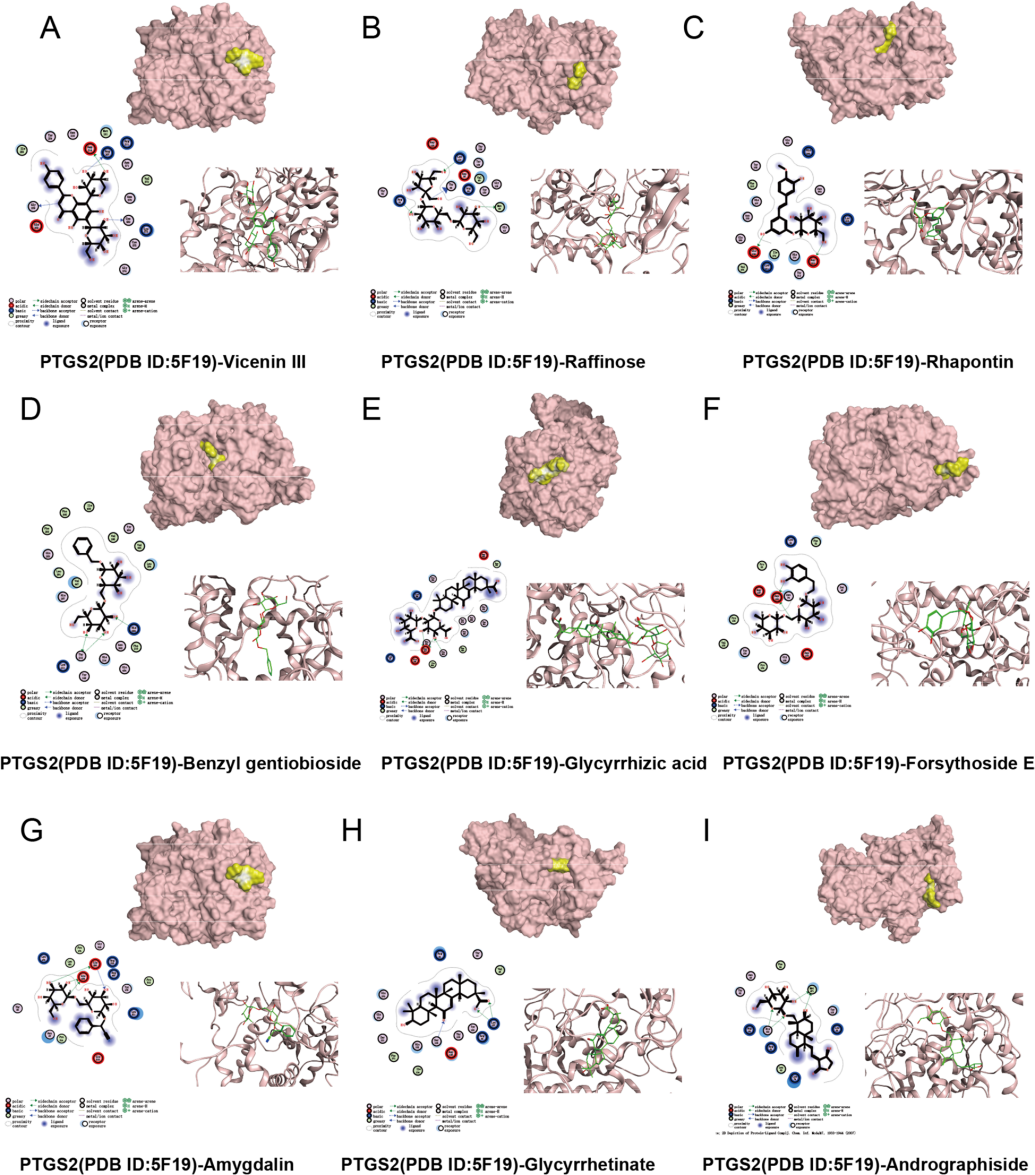
The molecular docking interactions between several active components of SRD and Prostaglandin-Endoperoxide Synthase 2(PTGS2). The 3D protein structures and ligand interaction diagrams are presented for each compound: (**A**) Vicenin III, (**B**) Raffinose, (**C**) Rhapontin, (**D**) Benzyl gentibioside, (**E**) Glycyrrhizic acid, (**F**) Forsythoside E, (**G**) Amygdalin, (**H**) Glycyrrhetinate, (**I**) Andrographiside

To validate the interactions between SRD's active compounds and UC-associated targets, molecular docking analysis was conducted. Compounds with binding affinities ≤ −5 kcal/mol were considered to exhibit strong binding potential. Of the 33 active components docked with PTGS2, 31 displayed favorable docking scores, with 27 scoring below −5 kcal/mol. The nine compounds with the highest docking scores included Vicenin III, Raffinose, Rhapontin, and others (Table 3). The molecular interactions and binding sites for these compounds are illustrated in Fig. 3A–I. These findings suggest that the active constituents of SRD may exert anti-inflammatory effects by inhibiting the expression of PTGS2, providing a molecular basis for SRD's therapeutic potential in the treatment of UC. However, further experimental validation is required to confirm these computational predictions and explore their clinical relevance.

**Table 3:** Highest rated in docking results

Compound	CAS	Protein (PDB ID)	Affinity (kcal/mol)
Vicenin III	185958	PTGS2(5F19)	− 7.4087
Raffinose	439242		− 7.2764
Rhapontin	637213		− 7.268
Benzyl gentiobioside	6453389		− 7.246
Glycyrrhizic acid	14982		− 7.235
Forsythoside E	69634125		− 7.0229
Amygdalin	656516		− 6.9074
Glycyrrhetinate	6857363		− 6.8645
Andrographiside	44593583		− 6.7765

### SRD alleviates DSS-induced colitis and enhances clinical and histopathological outcomes

**Fig. 4:**
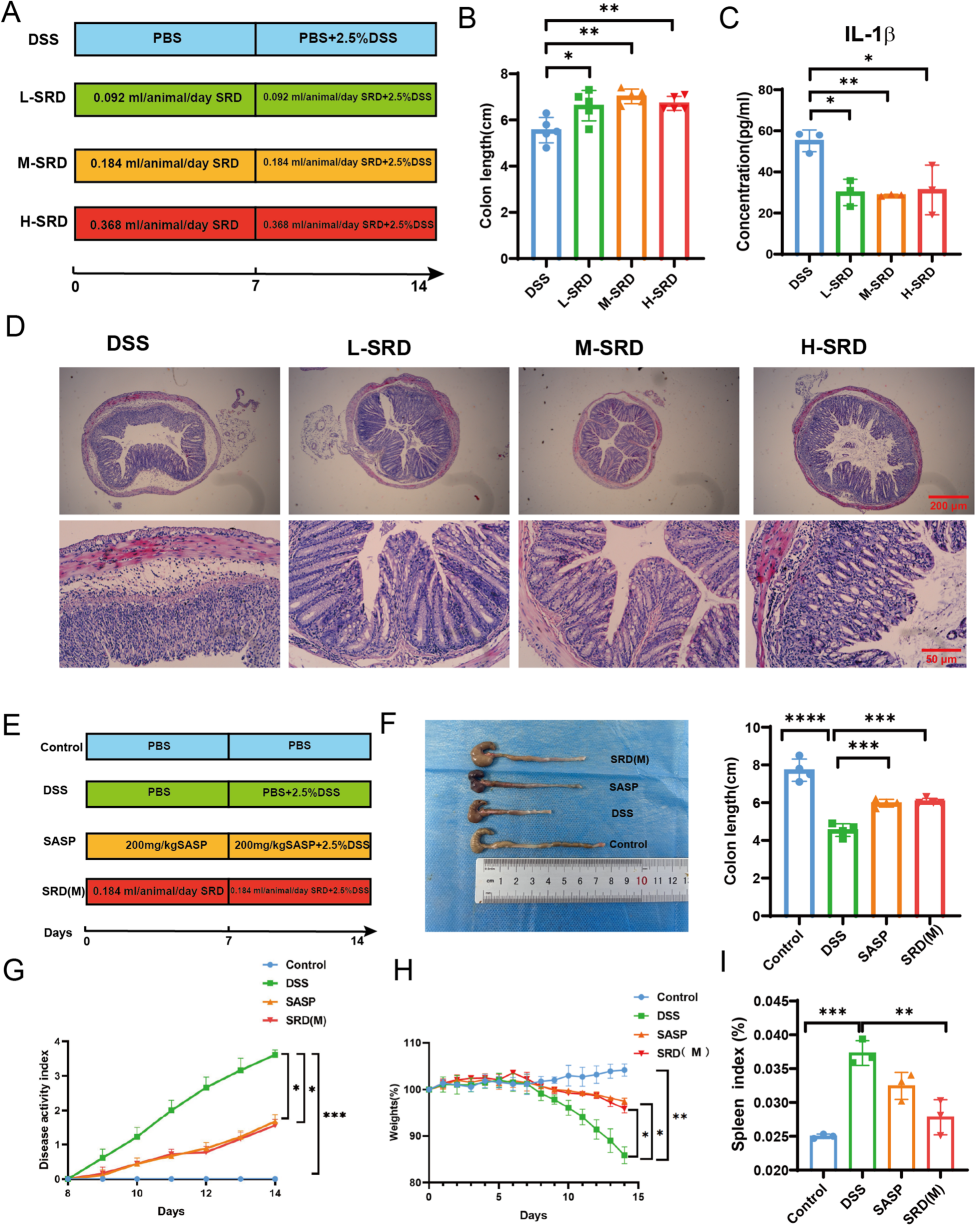
SRD Alleviates DSS-Induced ulcerative colitis in mice. **A** Experimental design for screening SRD concentrations against ulcerative colitis. **B** Comparison of colon length between DSS group and varying SRD dosage groups (high, medium, and low) (n = 5). **C** Serum expression levels of the inflammatory cytokine IL-1β across four experimental groups (n = 3). **D** Histological examination of colon tissue in ulcerative colitis mice via hematoxylin and eosin (H&E) staining. **E** Schematic representation of experimental grouping and treatment administration protocols for subsequent investigations. **F** Measurement of colon length in mice (n = 4). **G** Evaluation of the disease activity index (DAI) in the experimental cohorts. **H** Analysis of body weight changes throughout the experiment. **I** Assessment of the spleen index in experimental subjects (n = 3).Statistical significance indicated by *P < 0.05, **P < 0.01, ***P < 0.001, ****P < 0.0001

Animal experiments were conducted to optimize the dosage of SRD, focusing on colon length measurements (Fig. 4B), serum levels of IL-1β (Fig. 4C), and histological evaluation of HE-stained sections (Fig. 4D). The medium dose of SRD (SRD(M)) produced the most significant therapeutic effects and was selected for subsequent investigations.

**Fig. 5:**
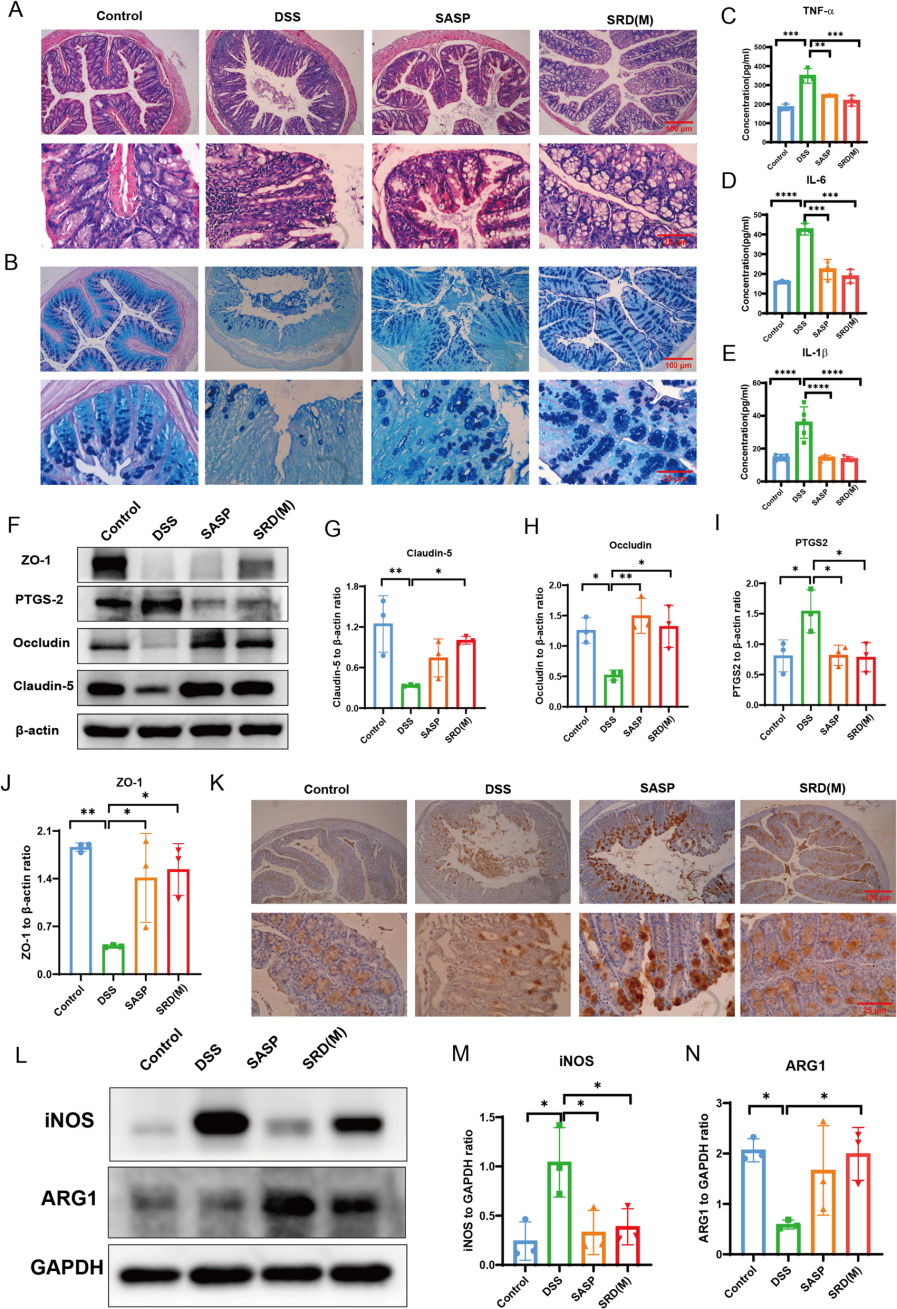
SRD restores intestinal barrier function and inhibits inflammatory factors in colitis mice. **A** Impact of SRD on H&E staining of the colon in mice with ulcerative colitis. **B** Alginate blue-periodic acid-Schiff(AB-PAS) staining images of mouse colon tissue. **C**–**E** Quantification of inflammatory factors, including TNF-α (n = 3), IL-6 (n = 3), and IL-1β (n = 5), in colon tissues across the four experimental groups. **F**–**J** Expression levels of Claudin-5, Occludin, ZO-1, and PTGS2 proteins in mouse colon tissue were assessed by immunoblotting. Results are presented as the mean ± SD (n = 3). **K** Immunohistochemical expression of Muc2 protein. **L**–**N** Expression levels of iNOS and ARG1 proteins in mouse colon tissue were assessed by immunoblotting. Results are presented as the mean ± SD (n = 3). Statistical significance indicated by *P < 0.05, **P < 0.01, ***P < 0.001, ****P < 0.0001

The efficacy of SRD(M) in a DSS-induced UC model was evaluated by assessing colon length, histopathology, and cytokine levels. Treatment with SRD(M) significantly increased colon length and reduced Disease Activity Index (DAI) scores, indicating a reduction in inflammation (Fig. 4F, G). Additionally, SRD(M) mitigated DSS-induced weight loss and reduced spleen weight, highlighting its protective effects (Fig. 4H, I). Histological examination revealed that SRD(M) alleviated epithelial damage, inflammatory cell infiltration, and crypt destruction (Fig. 5A). AB-PAS staining further demonstrated that SRD(M) preserved goblet cells, preventing DSS-induced mucosal barrier damage (Fig. 5B). ELISA results confirmed that SRD(M) significantly lowered the elevated levels of IL-1β, TNF-α, and IL-6 in colonic tissues (Fig. 5C–E), indicating a reduction in inflammation at the molecular level. Overall, these results suggest that SRD not only ameliorates the clinical manifestations of colitis but also promotes histological recovery and reduces inflammation.

### The Role of SRD in restoring intestinal barrier function in colitis mice

Tight junction (TJ) proteins, such as Claudin-5, Occludin, and ZO-1, are vital components of the intestinal mucosal barrier [27, 28]. They regulate intestinal permeability and play a crucial role in maintaining mucosal homeostasis. Western blot analysis revealed that TJ protein expression was significantly downregulated in the DSS group compared to the control group. However, SRD(M) and SASP treatment restored the expression of these proteins, indicating an improvement in intestinal barrier function. Additionally, PTGS2 expression was elevated in the DSS group compared to other treatment groups (Fig. 5F-J).

Muc2 mucin, a primary component of the mucus layer covering the colonic epithelium, plays a crucial role in protecting the intestinal barrier [29, 30]. Degradation of Muc2 compromises mucosal defense, making the epithelium more susceptible to bacterial invasion and exacerbating colitis [31–33]. Immunohistochemical analysis indicated a significant reduction in Muc2 expression following DSS treatment, which was reversed by SRD(M) and SASP treatments (Fig. 5K). These results suggest that SRD helps restore intestinal barrier function in UC by enhancing TJ protein expression and preserving the mucosal mucus layer.

### Impact of SRD on macrophage polarization

Macrophages are pivotal regulators of the immune response in inflammatory bowel disease (IBD), with M1-type macrophages (marked by CD86 and iNOS) promoting inflammation and tissue damage, whereas M2-type macrophages (characterized by CD163 and ARG1) contribute to anti-inflammatory responses and mucosal repair [14, 16, 34–38]. To investigate the impact of SRD(M) on macrophage polarization, both Western blotting and immunofluorescence analyses were performed.

Western blot results revealed that DSS treatment significantly upregulated the expression of iNOS, a representative marker of the M1 phenotype, while downregulating ARG1, a marker of the M2 phenotype. Notably, SRD(M) administration effectively reversed this pattern by suppressing iNOS levels and restoring ARG1 expression, suggesting a shift in macrophage polarization from a pro-inflammatory (M1) to an anti-inflammatory (M2) state (Fig. 5L–N).

**Fig. 6:**
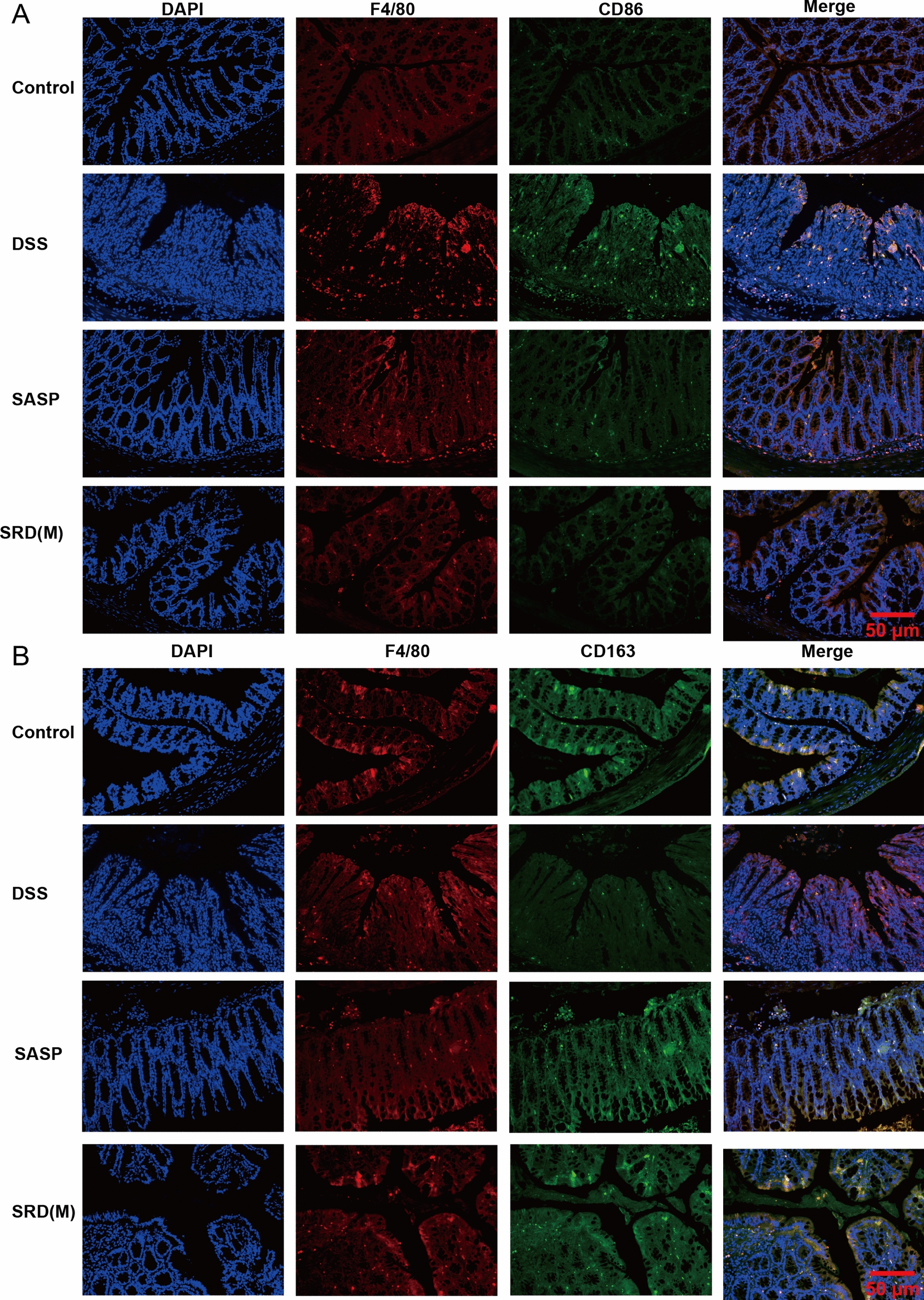
SRD facilitates the polarization of colonic macrophages from an M1 to an M2 phenotype in mice with ulcerative colitis. **A** Immunofluorescence staining images depicting M1 macrophages in mouse colon sections, with cell nuclei labeled using DAPI (blue). **B** Immunofluorescence staining images illustrating M2 macrophages in mouse colon sections

Consistently, immunofluorescence staining further demonstrated that DSS exposure led to elevated expression of CD86 in F4/80⁺ macrophages, indicating M1 polarization (Fig. 6A). In contrast, SRD(M) treatment reduced CD86⁺ macrophage infiltration and increased the number of CD163⁺ M2 macrophages (Fig. 6B). These data collectively support the notion that SRD(M) alleviates intestinal inflammation at least in part by modulating macrophage polarization, suppressing the pro-inflammatory M1 phenotype while promoting the reparative M2 subtype.

### Impact of SRD on gut microbiota composition

**Fig. 7:**
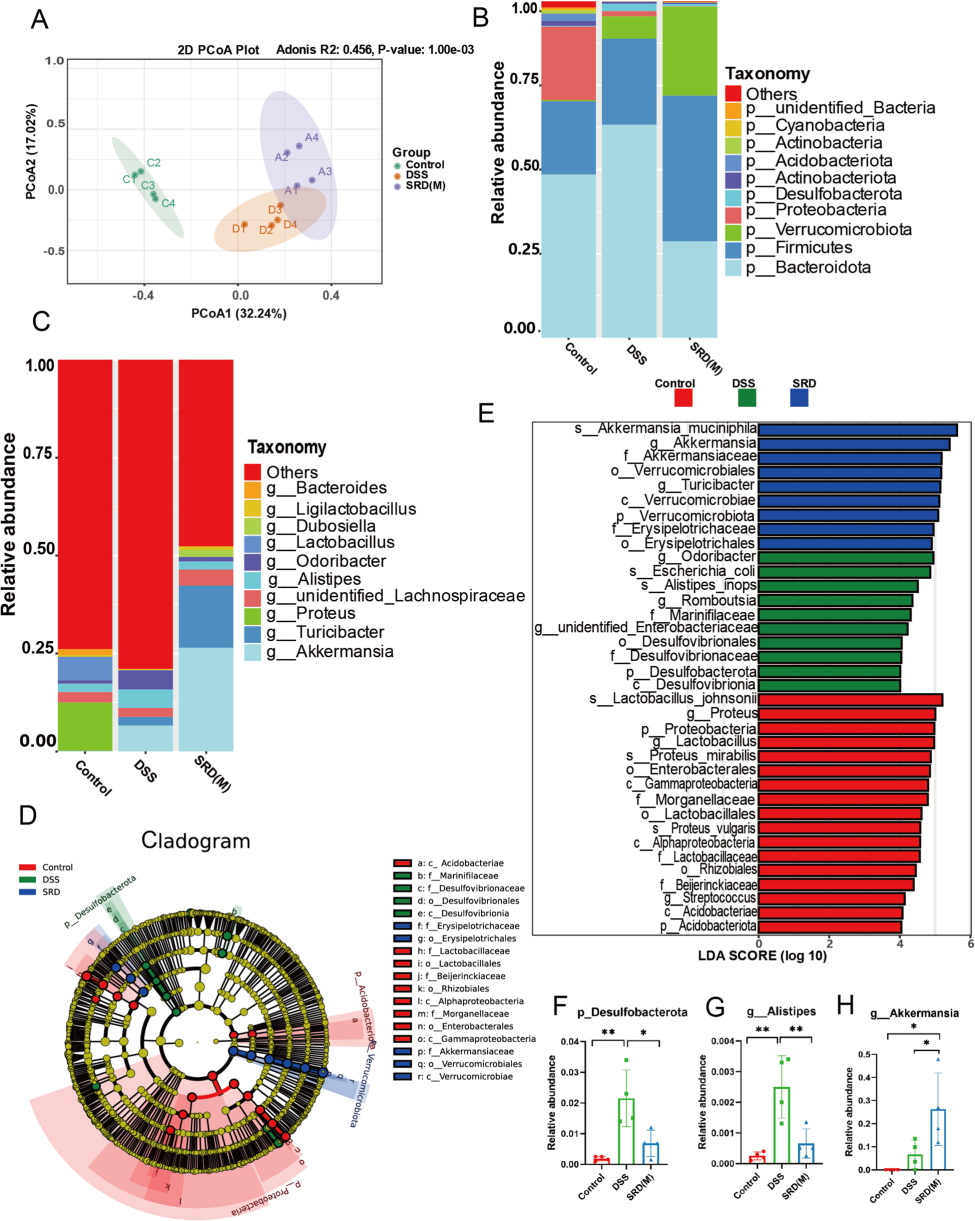
SRD(M) treatment mitigates DSS-induced gut microbiota dysbiosis. **A** Principal Coordinate Analysis (PCoA) plot showing distinct clustering of microbial communities in control, DSS, and SRD(M) groups based on beta diversity. **B** Gut microbial composition at the phylum level across the three groups of mice. **C** Differences in gut microbial composition at the genus level among the three groups of mice. **D** Cladogram based on LEfSe analysis showing community composition of the gut microbiota in mice. **E** Histogram of the LDA scores computed the differences in abundance between control, DSS, and SRD(M) groups. **F** Relative abundances of Desulfobacterota at the phylum level. **G**, **H** Relative abundances of Alistipes and Akkermansia at the genus level(n = 4). Statistical significance is indicated by *P < 0.05, **P < 0.01

The gut microbiota plays a crucial role in maintaining intestinal health, and its imbalance, or dysbiosis, is closely linked to the pathogenesis of UC [39]. To assess the impact of SRD on gut microbiota composition, we analyzed alpha diversity indices, as summarized in Table 4. Mice in the model group, treated with DSS, exhibited a significant decrease in both the Ace and Chao indices, accompanied by an increase in the Simpson index, compared to the control group. In contrast, SRD treatment increased both the Chao and Ace indices and reduced the Simpson index compared to the model group. These results suggest that SRD treatment improved the richness and diversity of the gut microbiota in DSS-induced colitis mice, indicating its potential to regulate intestinal microbiota composition. Principal Coordinates Analysis (PCoA) confirmed significant differences between the DSS, SRD(M), and control groups (Fig. 7A).

**Table 4:** Alpha diversity parameters assessed by the Simpson, Chao1, and ACE indices

Group	Simpson	Chao1	ACE
Control	0.927	1942.71225	2091.235
DSS	0.951	342.18	349.35
SRD	0.833	793.319	880.368

At the phylum level, DSS treatment resulted in a marked increase in Desulfobacterota, a sign of dysbiosis associated with inflammation. SRD(M) treatment reversed this effect, restoring the relative abundances of Bacteroidota and Firmicutes to levels similar to those of the control group while also reducing the overgrowth of Desulfobacterota (Fig. 7B). At the genus level, DSS administration significantly increased the abundance of Alistipes, a genus associated with inflammation. In contrast, SRD(M) treatment attenuated this increase. Additionally, the beneficial genus Akkermansia was more abundant in the SRD(M) group than in either the DSS or control groups, suggesting its role in maintaining gut homeostasis (Fig. 7C).

LEfSe analysis identified key bacterial taxa that differed significantly among the groups (Fig. 7D), with Akkermansia showing marked enrichment in the SRD(M) group (Fig. 7E). Analysis of specific bacterial taxa revealed that DSS treatment increased Desulfobacterota abundance, which SRD(M) partially mitigated (Fig. 7F). Similarly, Alistipes was elevated in the DSS group but reduced following SRD(M) treatment (Fig. 7G). At the same time, Akkermansia was notably enriched in the SRD(M) group (Fig. 7H). These results suggest that SRD exerts a modulatory effect on the gut microbiota, promoting the growth of beneficial microbes while suppressing the growth of harmful taxa.

### Impact of SRD on metabolites and their correlations with gut microbiota

**Fig. 8:**
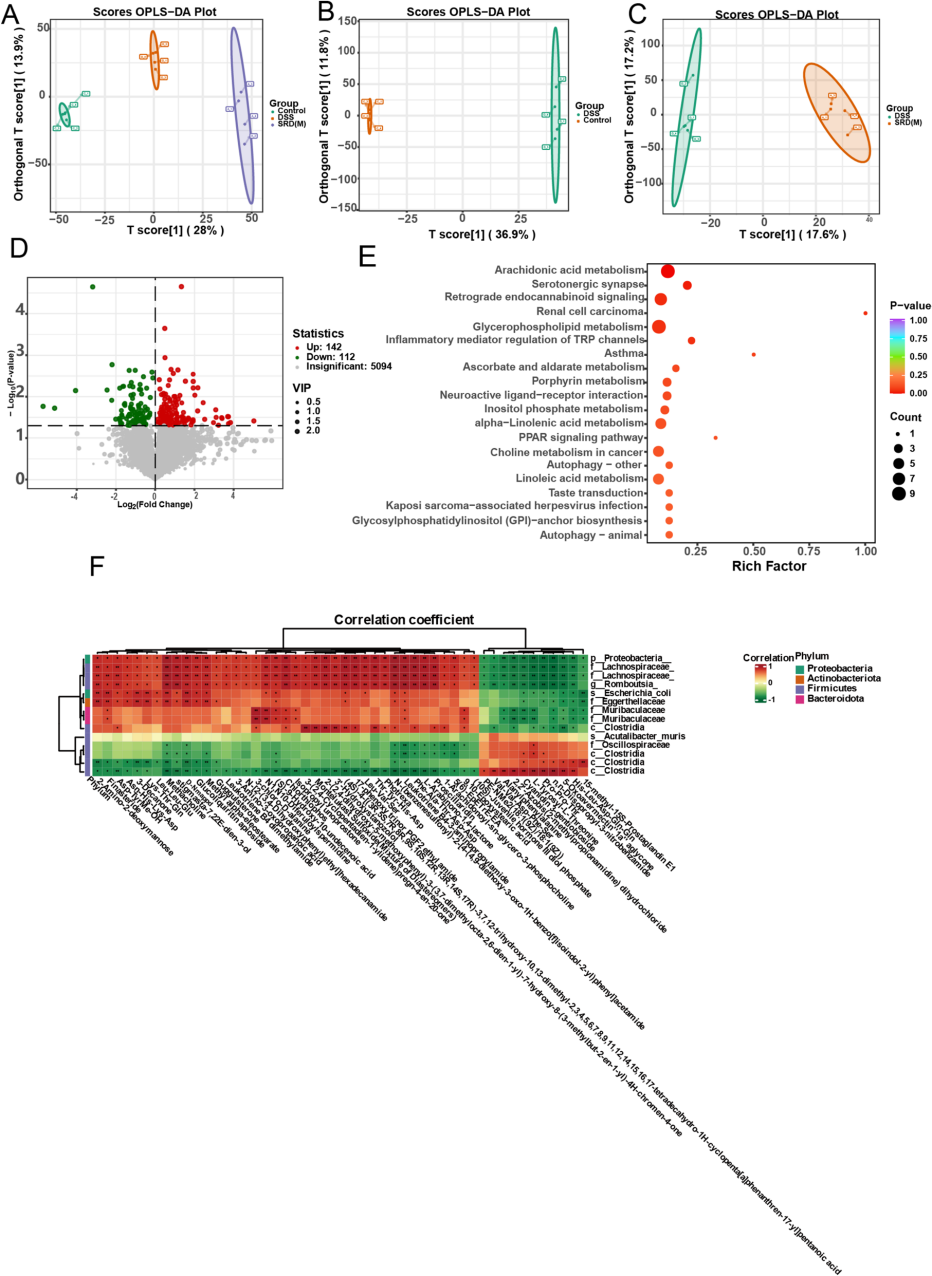
Metabolomic analysis of cecal contents. **A**–**C** Orthogonal Partial Least Squares Discriminant Analysis (OPLS-DA) score plots illustrate distinct separations between the control group (orange) and the experimental groups (green and purple). **D** Volcano plot of differential metabolites, where red dots represent significantly upregulated metabolites, green dots indicate significantly downregulated metabolites, and grey dots denote non-significant changes. **E** KEGG enrichment analysis of differential metabolites between the SRD and DSS groups. **F** Correlation analysis between metabolites and gut microbiota, with bacterial species represented in rows and metabolites in columns. The color scale, from green to red, indicates negative and positive correlations, respectively

Non-targeted metabolomics analysis of intestinal contents revealed significant differences among the three mouse groups. OPLS-DA in positive ion mode demonstrated clear separation, particularly between the DSS and control, as well as the SRD(M) and DSS groups (Fig. 8A–C). Cluster analysis identified 254 differential metabolites, with 142 upregulated and 112 downregulated (Fig. 8D).

KEGG pathway enrichment analysis revealed significant changes, particularly in arachidonic acid metabolism, highlighting its role in inflammatory regulation and aligning with SRD’s anti-inflammatory effects. Other affected pathways included the serotonergic synapse and retrograde endocannabinoid signaling, indicating SRD’s influence on neural and lipid signaling (Fig. 8E). These findings suggest that SRD modulates gut microbiota-driven metabolic pathways, thereby enhancing inflammatory regulation and potentially conferring neuroprotective effects.

Correlation analysis revealed that Guggulsterone, an anti-inflammatory metabolite, was positively associated with Firmicutes, such as Lachnospiraceae and Romboutsia, suggesting SRD may promote these bacteria to boost Guggulsterone production. Similarly, Cyanidin 3-gentiobioside correlated with Oscillospiraceae and Clostridia, indicating their potential role in its metabolism (Fig. 8F). Overall, Firmicutes play a crucial role in modulating anti-inflammatory metabolites during SRD treatment.

### Fecal microbiota transplantation (FMT) and the therapeutic role of gut microbiota in SRD treatment

**Fig. 9:**
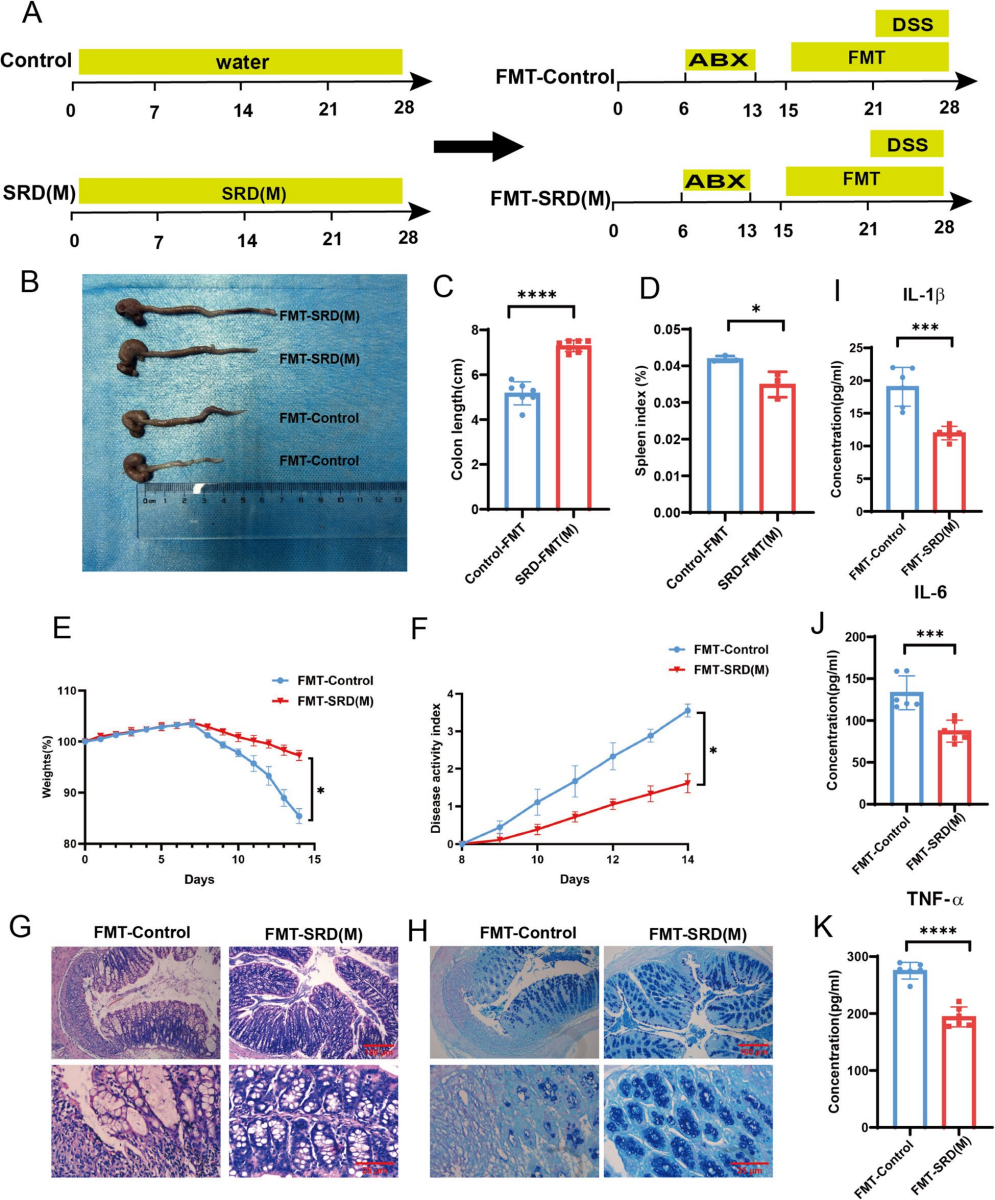
FMT alleviates the severity of DSS-induced UC in mice. **A** Experimental design. **B** Representative images of mouse colons on day 29. **C** Colon length of mice (n = 7). **D** Spleen index (n = 3). **E** Body weight changes during the experiment. **F** Disease activity index (DAI). **G** H&E-stained images of mouse colon tissue. **H** AB-PAS stained images of mouse colon tissue. **I**–**K** Expression levels of inflammatory cytokines, including TNF-α, IL-6, and IL-1β, in mouse colon tissue (n = 6). Differences are considered statistically significant at* P* < 0.05. *P < 0.05, **P < 0.01, ***P < 0.001, ****P < 0.0001

To evaluate the role of gut microbiota in mediating the therapeutic effects of SRD in colitis, fecal microbiota transplantation (FMT) was performed using fecal suspensions from donor mice. Recipient mice treated with FMT-SRD(M) exhibited significantly less colon shortening compared to those in the FMT-Control group (Fig. 9B, C). Additionally, the FMT-Control group showed marked body weight loss, an elevated spleen index, and higher disease activity index (DAI) scores, all of which were significantly ameliorated in the FMT-SRD(M) group (Fig. 9D–F).

Histopathological evaluation revealed that FMT-SRD(M) treatment attenuated epithelial damage, inflammatory cell infiltration, and crypt loss, while notably increasing goblet cell numbers (Fig. 9G, H). Consistently, pro-inflammatory cytokine levels were significantly reduced in the FMT-SRD(M) group, further supporting its anti-inflammatory efficacy (Fig. 9I–K).

**Fig. 10:**
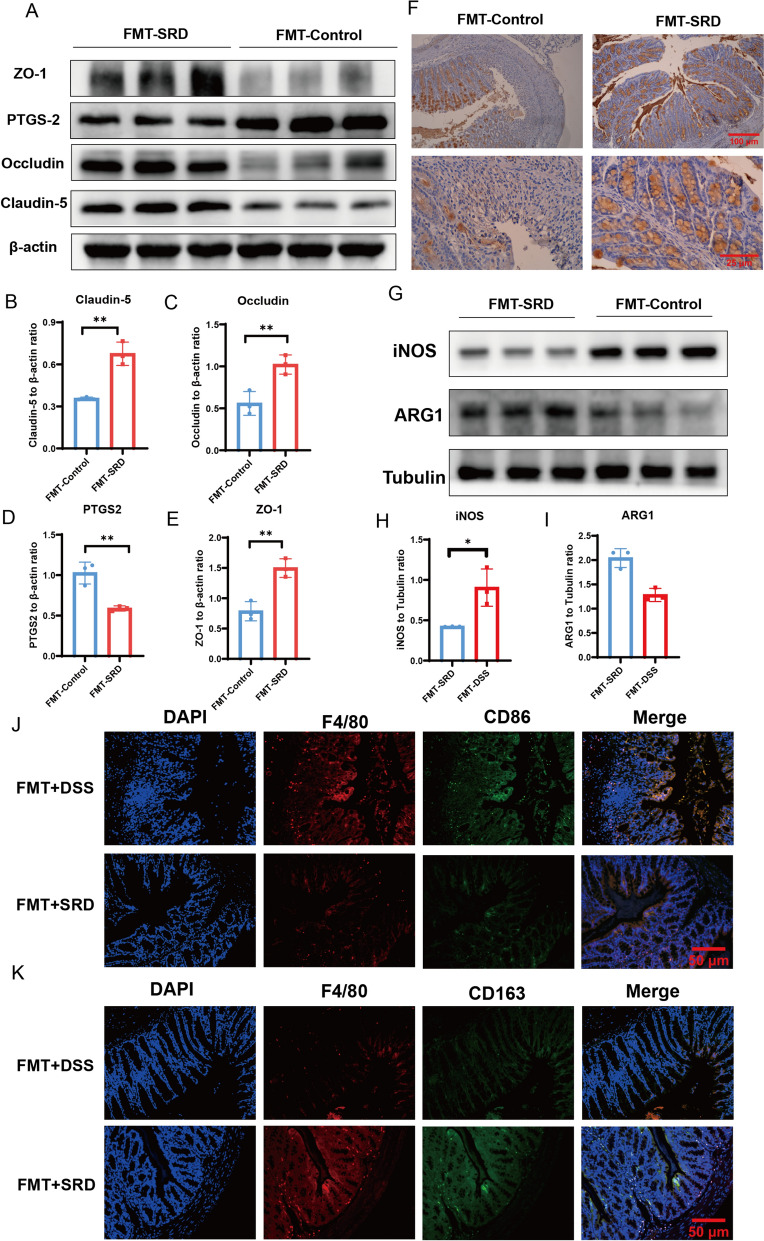
FMT enhances intestinal barrier integrity and facilitates the polarization of M2 macrophages. **A**–**E** Expression of Claudin-5, Occludin, ZO-1, and PTGS2 proteins in mouse colonic tissue by WB (n = 3). **F** Expression of Muc2 protein by immunohistochemistry. **G**–**I** Expression of iNOS and ARG1proteins in mouse colonic tissue by WB (n = 3). **J** Immunofluorescence staining of M1 macrophages in mouse colonic sections. **K** Immunofluorescence staining of M2 macrophages in mouse colonic sections. Statistical significance indicated by *P < 0.05, **P < 0.01, ***P < 0.001, ****P < 0.0001

At the molecular level, Western blot analysis showed upregulation of tight junction proteins, including Claudin-5, Occludin, and ZO-1, in the FMT-SRD(M) group, suggesting enhanced intestinal barrier integrity. Conversely, PTGS2 expression remained elevated in the FMT-Control group, indicating ongoing inflammation and impaired resolution capacity (Fig. 10A–E). Immunohistochemical staining of Muc-2 further confirmed improved mucosal protection in the FMT-SRD(M) group (Fig. 10F).

Moreover, SRD(M) influenced macrophage polarization in the colonic tissue. Western blot analysis revealed higher iNOS expression in the FMT-Control group, indicative of M1-type pro-inflammatory macrophage activation, whereas ARG1 expression—a marker of M2-type anti-inflammatory macrophages—was significantly upregulated in the FMT-SRD(M) group (Fig. 10G–I). These findings were corroborated by double immunofluorescence staining, which demonstrated increased CD86⁺ M1 macrophages in the FMT-Control group and elevated CD163⁺ M2 macrophages in the FMT-SRD(M) group (Fig. 10J, K).

**Fig. 11:**
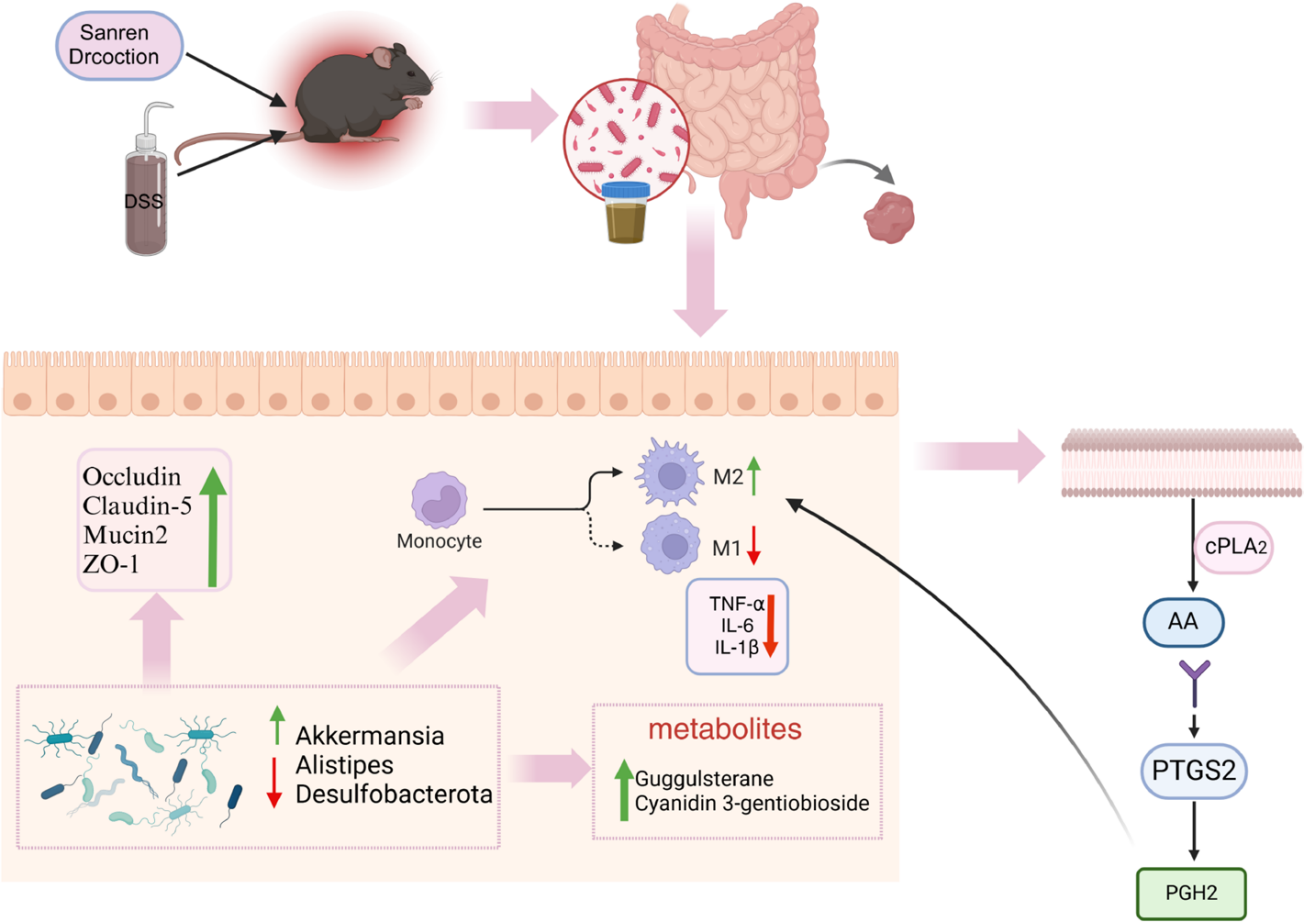
The schematic diagram of the mechanism by which SRD alleviates DSS-induced ulcerative colitis. SRD, Sanren decoction; DSS, Dextran sulfate sodium; cPLA2, Cytosolic phospholipase A2; AA, Amino acid; PTGS2, Prostaglandin-endoperoxide synthase 2; PGH2, Prostaglandin H2

Taken together, these results demonstrate that the therapeutic benefits of SRD are intimately linked to its ability to reshape the gut microbiota, strengthen epithelial barrier integrity, modulate immune responses, and drive macrophage polarization toward an anti-inflammatory state, ultimately facilitating the restablishment of intestinal homeostasis Fig. 11.

## Discussion

This study sheds light on the pharmacological mechanisms by which Sanren Decoction (SRD) exerts therapeutic effects against ulcerative colitis (UC). By employing a network pharmacology approach, we identified 87 common target genes that link SRD's active compounds to the pathology of UC, with PTGS2 identified as a central target. This is noteworthy since COX-2 is recognized in the existing literature as a crucial mediator of inflammatory responses, especially in gastrointestinal disorders [40]. The strong binding affinity between several SRD compounds and PTGS2 highlights their potential to influence inflammatory pathways, indicating a targeted mechanism for alleviating UC symptoms.

Our pathway enrichment analysis revealed that the identified targets are significantly involved in inflammation-related pathways, particularly those associated with cytokine signaling. This is important because UC is characterized by an exacerbated inflammatory response driven by pro-inflammatory cytokines, such as IL-1β and TNF-α [41]. The therapeutic effects of SRD, demonstrated in our animal studies, align with these pathways. The medium dose of SRD (SRD(M)) not only significantly improved colon length and reduced the Disease Activity Index (DAI) scores, reflecting decreased inflammation, but also supported histological recovery. Histopathological evaluations revealed improved epithelial integrity and reduced inflammatory cell infiltration, reinforcing previous studies that emphasize the restoration of mucosal health in the treatment of UC.

In examining intestinal barrier function, we found restoration of tight junction proteins (TJ proteins), such as Claudin-5, Occludin, and ZO-1, which are essential for maintaining intestinal permeability and mucosal integrity. The significant reduction of TJ protein expression in DSS-induced colitis models corroborates findings from prior research that highlight compromised intestinal barrier function as a key factor in UC pathogenesis. SRD treatment not only restored these proteins but also downregulated PTGS2 expression, suggesting a dual action of SRD in reducing inflammation and enhancing barrier integrity. This multifaceted approach is crucial as it addresses both inflammation and barrier dysfunction, two significant aspects of UC.

Another significant focus of this study was the exploration of macrophage polarization. The shift from a pro-inflammatory M1 macrophage phenotype to an anti-inflammatory M2 phenotype after SRD treatment underscores its potential to modulate immune responses. This is particularly relevant given the role of macrophages in UC, where M1 macrophages can exacerbate tissue damage, while M2 macrophages facilitate tissue repair. Our findings support the existing literature, which suggests that promoting M2 polarization could be an effective therapeutic strategy in UC. SRD’s ability to influence macrophage polarization highlights its role as an anti-inflammatory agent that targets not only cytokines but also the cellular environment contributing to inflammation.

The impact of SRD on gut microbiota composition represents another vital contribution of this research. Our analyses indicated that SRD treatment effectively restored the gut microbiota balance disrupted by DSS. The increase in beneficial bacteria, especially Akkermansia, emphasizes SRD’s role in promoting gut homeostasis. Akkermansia is associated with improved gut barrier function and reduced inflammation [42], further validating SRD's therapeutic potential. Additionally, the changes in gut microbiota metabolites, as revealed by our non-targeted metabolomics analysis, suggest a complex interaction among SRD, gut microbiota, and metabolic pathways. Identifying 254 differential metabolites illustrates the extensive impact of SRD, particularly in lipid signaling and inflammatory regulation. The positive correlation between Guggulsterone and Firmicutes [43] indicates that SRD not only encourages beneficial bacteria but also enhances the production of anti-inflammatory metabolites.

Importantly, our results demonstrated that the SRD-induced modulation of gut microbiota is functionally linked to immune regulation, particularly macrophage polarization. The concurrent shift in gut microbial composition and the increase in M2 macrophage markers suggest a coordinated response, wherein changes in microbiota contribute to reprogramming mucosal immune responses. FMT experiments further validated this relationship.

In the FMT assays, recipient mice receiving microbiota from SRD-treated donors exhibited not only reduced clinical signs of colitis and improved histopathology but also enhanced expression of tight junction proteins and a significant shift from M1 to M2 macrophages in colonic tissues. Furthermore, levels of inflammatory cytokines, such as TNF-α and IL-1β, were decreased, indicating that the microbiota transferred from SRD-treated mice retained their functional immunomodulatory capacity. These findings provide strong evidence that SRD’s therapeutic effects are mediated, at least in part, through a microbiota–macrophage–inflammation axis, highlighting the gut microbiota as an active driver of mucosal immune regulation rather than a passive responder.

Together, these data propose a mechanistic framework in which SRD first reshapes gut microbiota, which in turn modulates macrophage polarization toward an anti-inflammatory M2 phenotype, thereby dampening intestinal inflammation and promoting mucosal healing. This integrated interaction between the microbiota and host immune system underscores the systemic impact of SRD and supports its multi-target therapeutic potential.

Compared with conventional UC therapies such as 5-aminosalicylic acid, corticosteroids, or biologic agents targeting single inflammatory pathways, SRD demonstrates a broader therapeutic profile. Its effects span multiple biological systems—including inflammatory signaling, epithelial barrier repair, macrophage polarization, and gut microbiota modulation—suggesting a more integrated and potentially holistic mechanism of action. This multi-target approach may be particularly advantageous in addressing the complex and relapsing nature of UC, which often involves dysregulation across multiple axes, including the immune, epithelial, and microbial systems.

However, this study has certain limitations. While network pharmacology and molecular docking analyses provided valuable insights into potential targets and mechanisms, these methods are primarily predictive and require further experimental validation to confirm their findings. Additionally, the DSS-induced colitis model in mice, though useful, may not fully capture the complexity of UC in humans. Future research should include clinical trials to validate these findings and explore the long-term efficacy and safety of SRD in UC patients. Moreover, as the DSS-induced colitis model primarily reflects acute inflammation rather than the chronic course of human UC, further validation in chronic or immune-mediated colitis models will be necessary to confirm the translational relevance of these findings.

In conclusion, this study provides a comprehensive analysis of the pharmacological mechanisms of SRD in UC, demonstrating its multi-target effects on inflammation, intestinal barrier function, macrophage polarization, gut microbiota regulation, and metabolic pathways. These findings enhance the understanding of SRD's therapeutic potential and provide a scientific foundation for its clinical application. To further advance this research, future studies will include well-designed clinical trials to evaluate the efficacy and safety of SRD in patients with UC. In addition, mechanistic studies using cell-specific approaches, microbiota–host interaction models, and metabolomic profiling will be conducted to clarify the molecular pathways underlying SRD’s effects.

## Supplementary Information

Supplementary Material 1

## Data Availability

The data associated with this study can be obtained from the corresponding author upon reasonable request.

## Abbreviations

AA: Arachidonic acid
cPLA2: Cytosolic phospholipase A2
PTGS2: Prostaglandin-endoperoxide synthase 2
PGH2: Prostaglandin H2
AB-PAS: Alcian blue-periodic acid-Schiff
COX-2: Cyclooxygenase-2
DAI: Disease activity index
DSS: Dextran sodium sulfate
ELISA: Enzyme-linked immunosorbent assay
FMT: Fecal transplantation
H&E: Hematoxylin and eosin
IBD: Inflammatory bowel disease
IL-1β: Interleukin 1β
IL-6: Interleukin 6
TNF-α: Tumor necrosis factor-α
LC–MS: Liquid chromatography-tandem mass spectrometry
LDA: Linear discriminant analysis
LEfSe: Linear discriminant analysis effect size
MUC-2: Mucin-2
OPLS-DA: Orthogonal partial least squares discriminant analysis
OTUs: Operational taxonomic units
PBS: Phosphate-buffered saline
PCA: Principal component analysis
PCoA: Principal coordinate analysis
PLA2: Phospholipase A2
PPI: Protein–protein interaction
SASP: Sulfasalazine
SD: Standard deviation
TJ: Tight junction
TNF-α: Tumor necrosis factor-α
UC: Ulcerative colitis
SRD: Sanren Decoction
KEGG: Kyoto encyclopedia of genes and genomes
DAPI: 4′,6-diamidino-2-phenylindole
WB: Western blotting
