# Supplementary figures and images for: Sanren decoction ameliorates ulcerative colitis by modulating gut microbiota and macrophage polarization to enhance intestinal barrier function
Source: Chin Med. 2025 Aug 20;20:128. doi: 10.1186/s13020-025-01183-1 (PMC12366350; doi:10.1186/s13020-025-01183-1)

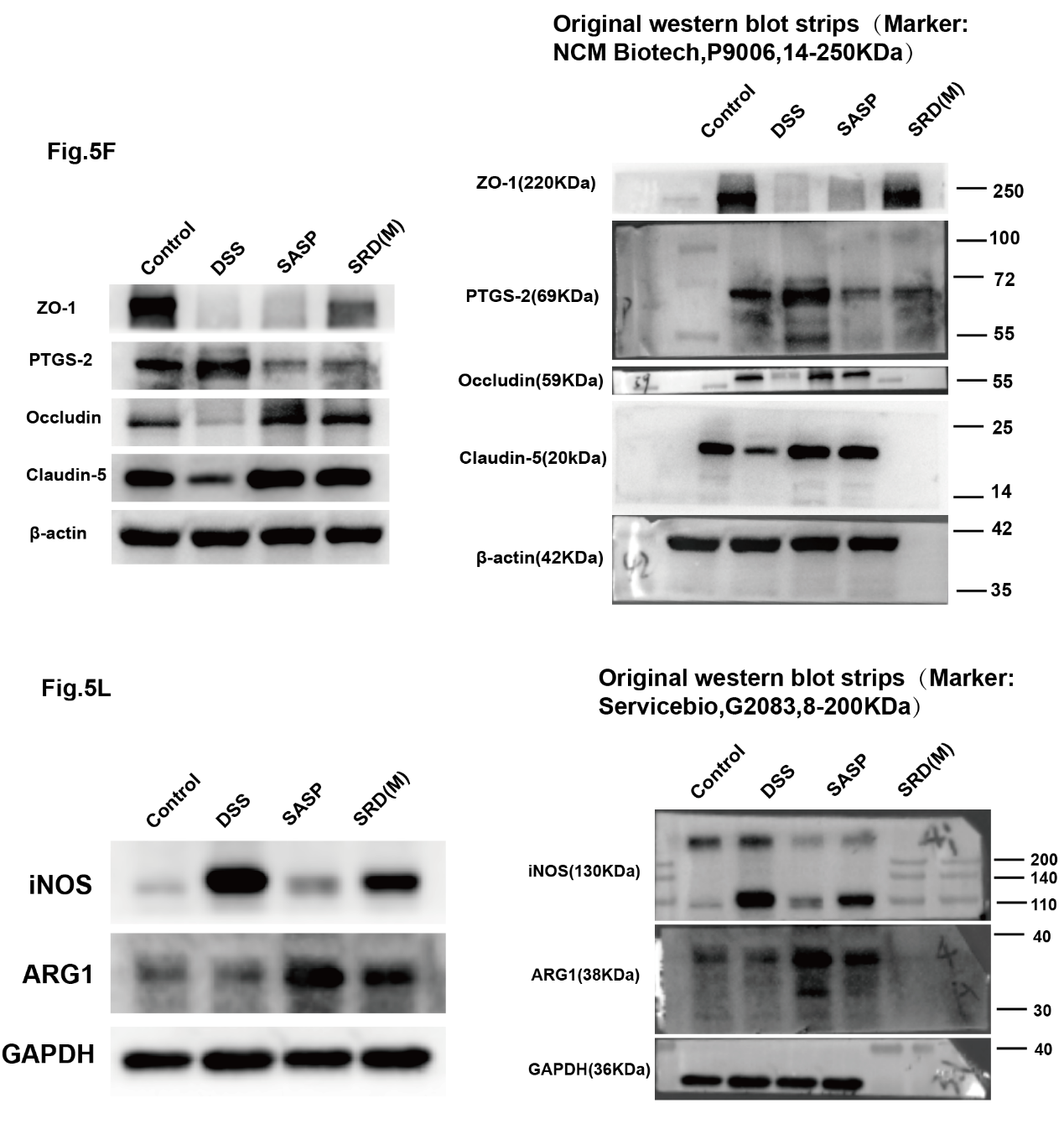


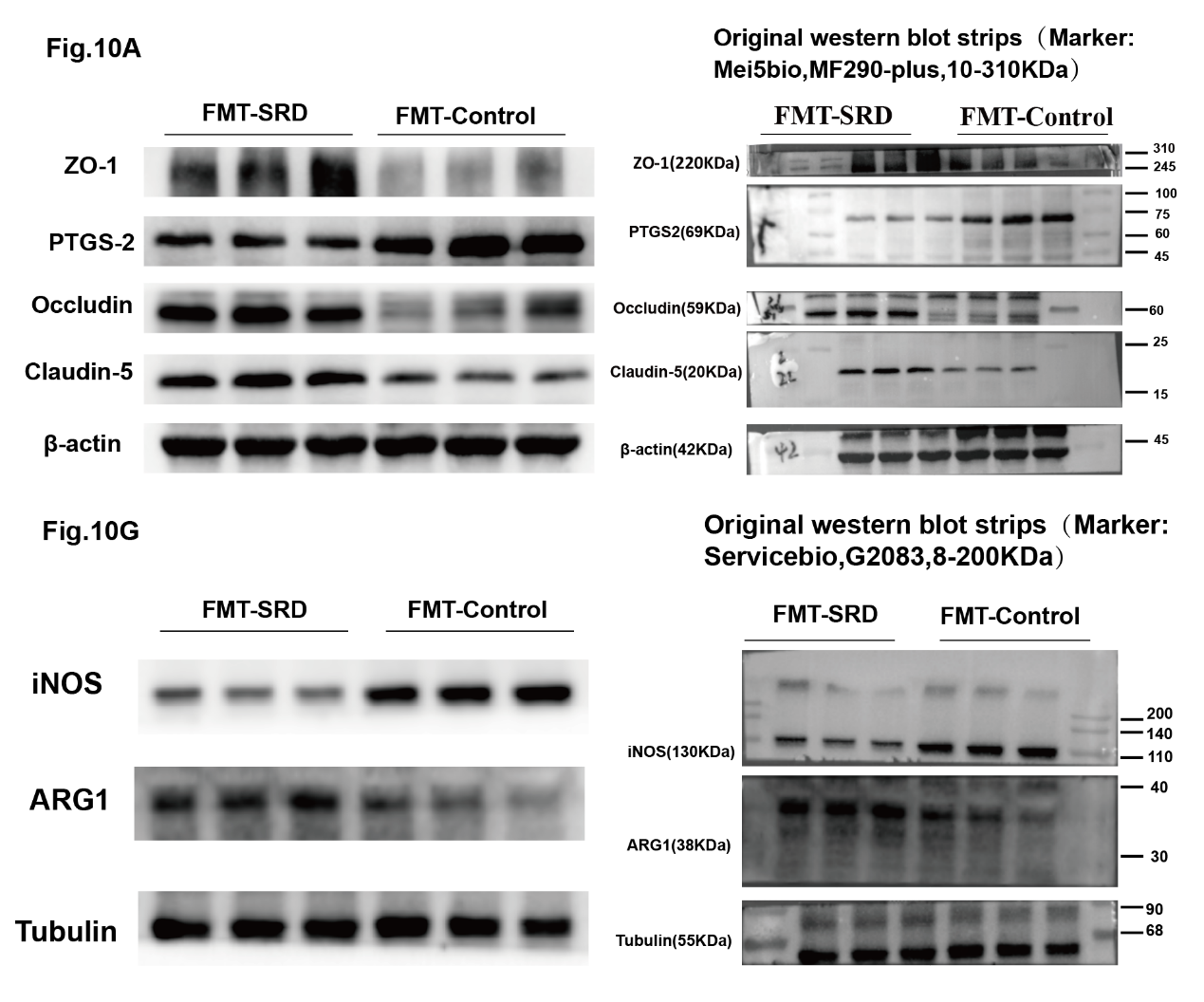


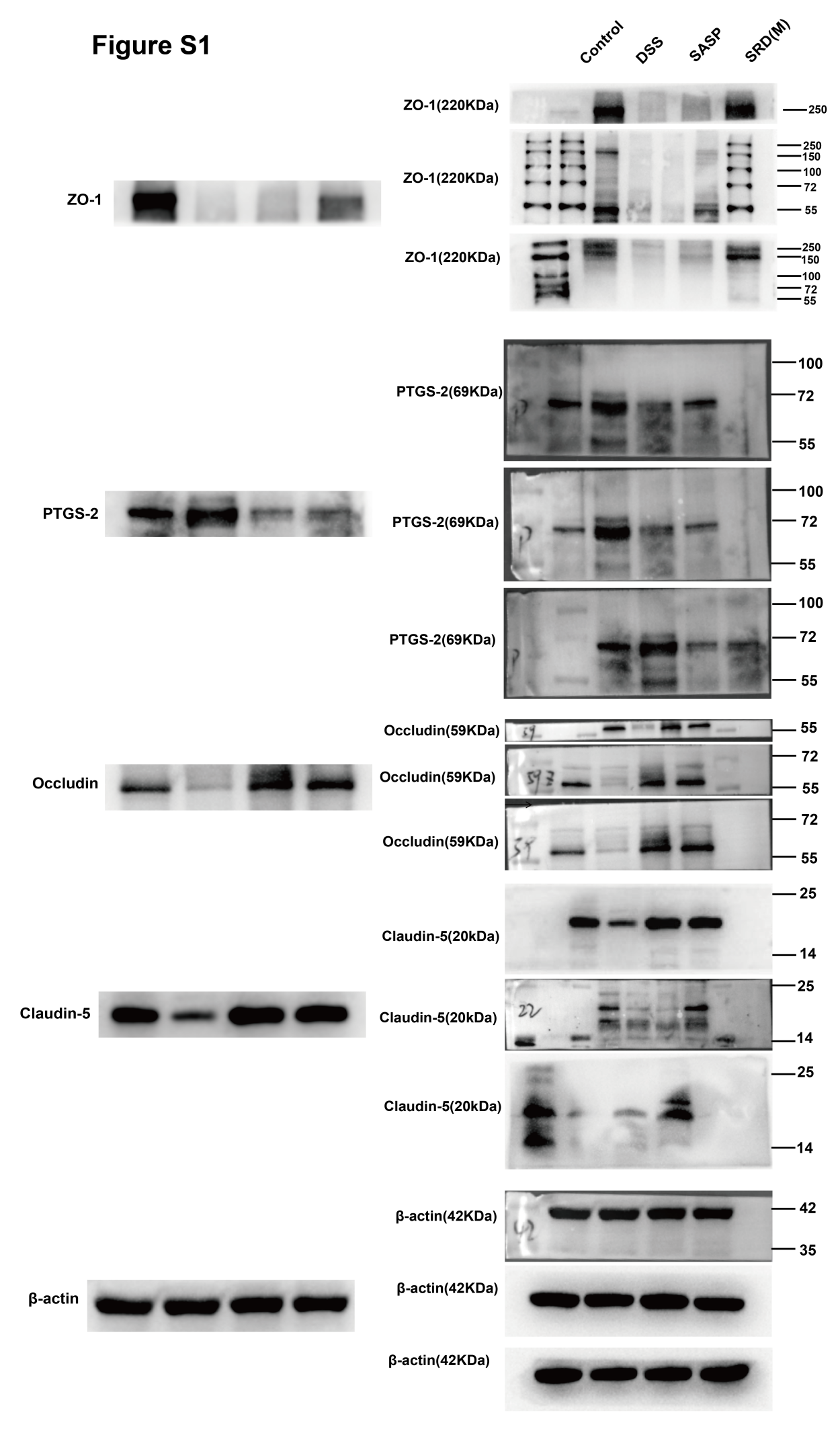


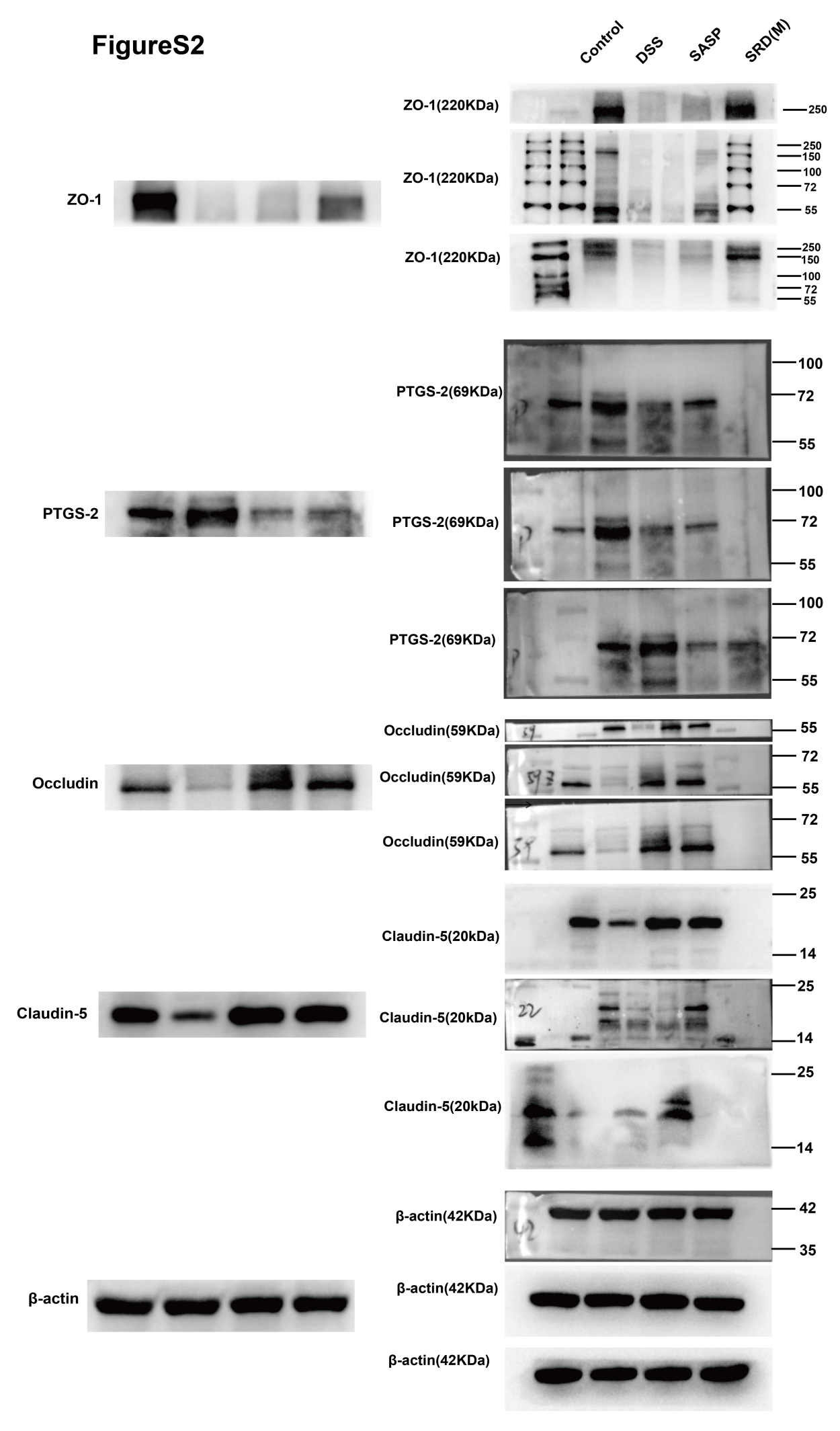


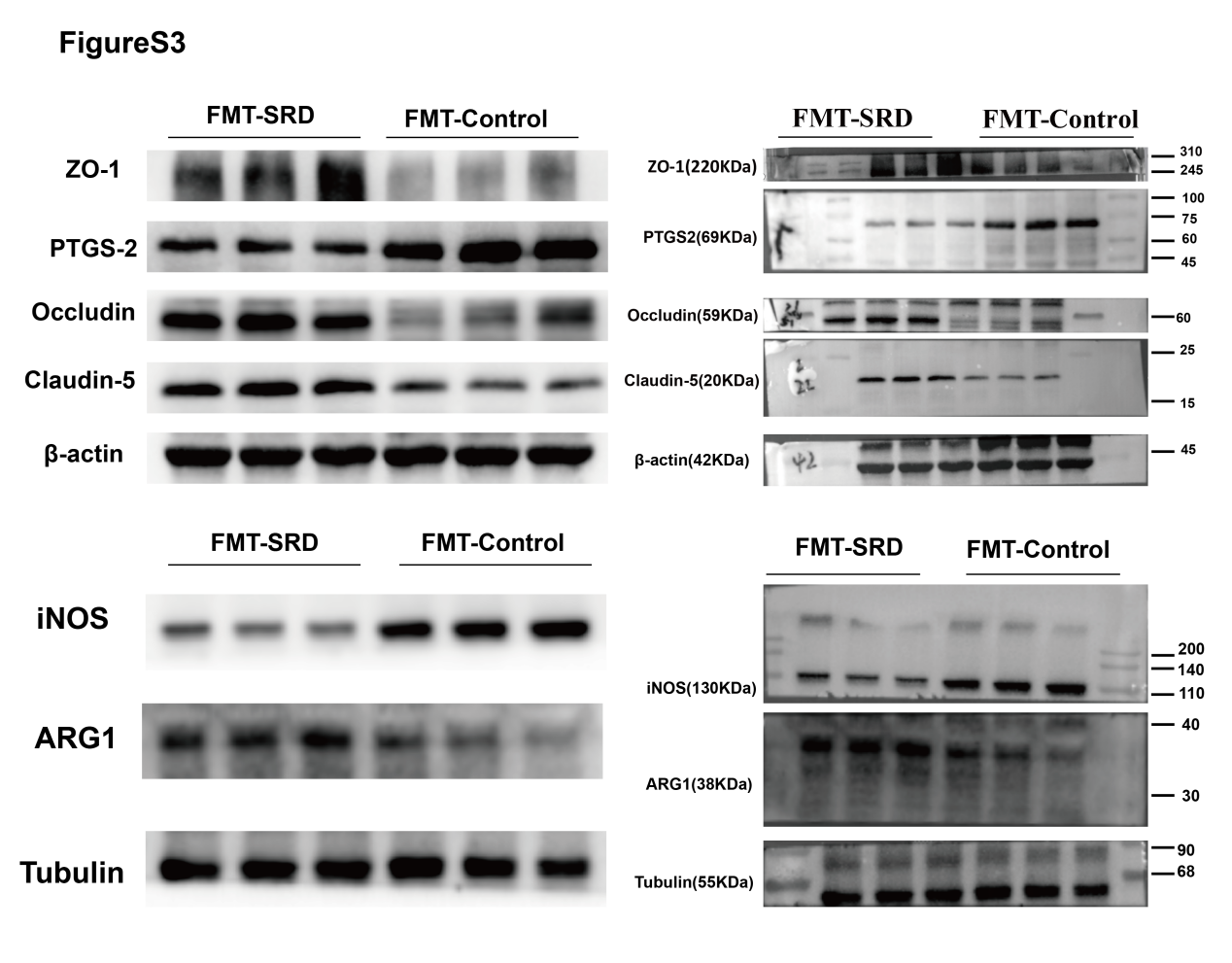

Supplement: Supplementary file 1 — Supplementary Material 1 [file 13020_2025_1183_MOESM1_ESM.docx]
